# Transit Time Measurement in Indicator Dilution Curves: Overcoming the Missing Ground Truth and Quantifying the Error

**DOI:** 10.3389/fphys.2021.588120

**Published:** 2021-05-28

**Authors:** Ady Naber, Michael Reiß, Werner Nahm

**Affiliations:** Institute of Biomedical Engineering, Karlsruhe Institute of Technology, Karlsruhe, Germany

**Keywords:** indicator dilution curve, mathematical fits, transit time, blood flow velocity, fluorescence angiography, sub-frame rate accuracy

## Abstract

The vascular function of a vessel can be qualitatively and intraoperatively checked by recording the blood dynamics inside the vessel via fluorescence angiography (FA). Although FA is the state of the art in proving the existence of blood flow during interventions such as bypass surgery, it still lacks a quantitative blood flow measurement that could decrease the recurrence rate and postsurgical mortality. Previous approaches show that the measured flow has a significant deviation compared to the gold standard reference (ultrasonic flow meter). In order to systematically address the possible sources of error, we investigated the error in transit time measurement of an indicator. Obtaining *in vivo* indicator dilution curves with a known ground truth is complex and often not possible. Further, the error in transit time measurement should be quantified and reduced. To tackle both issues, we first computed many diverse indicator dilution curves using an *in silico* simulation of the indicator's flow. Second, we post-processed these curves to mimic measured signals. Finally, we fitted mathematical models (parabola, gamma variate, local density random walk, and mono-exponential model) to re-continualize the obtained discrete indicator dilution curves and calculate the time delay of two analytical functions. This re-continualization showed an increase in the temporal accuracy up to a sub-sample accuracy. Thereby, the Local Density Random Walk (LDRW) model performed best using the cross-correlation of the first derivative of both indicator curves with a cutting of the data at 40% of the peak intensity. The error in frames depends on the noise level and is for a signal-to-noise ratio (SNR) of 20 *dB* and a sampling rate of *f*_*s*_ = 60 *Hz* at $f_{s}^{-1}\cdot 0.25\left(\pm 0.18\right)$, so this error is smaller than the distance between two consecutive samples. The accurate determination of the transit time and the quantification of the error allow the calculation of the error propagation onto the flow measurement. Both can assist surgeons as an intraoperative quality check and thereby reduce the recurrence rate and post-surgical mortality.

## 1. Introduction

**Figure 1:**
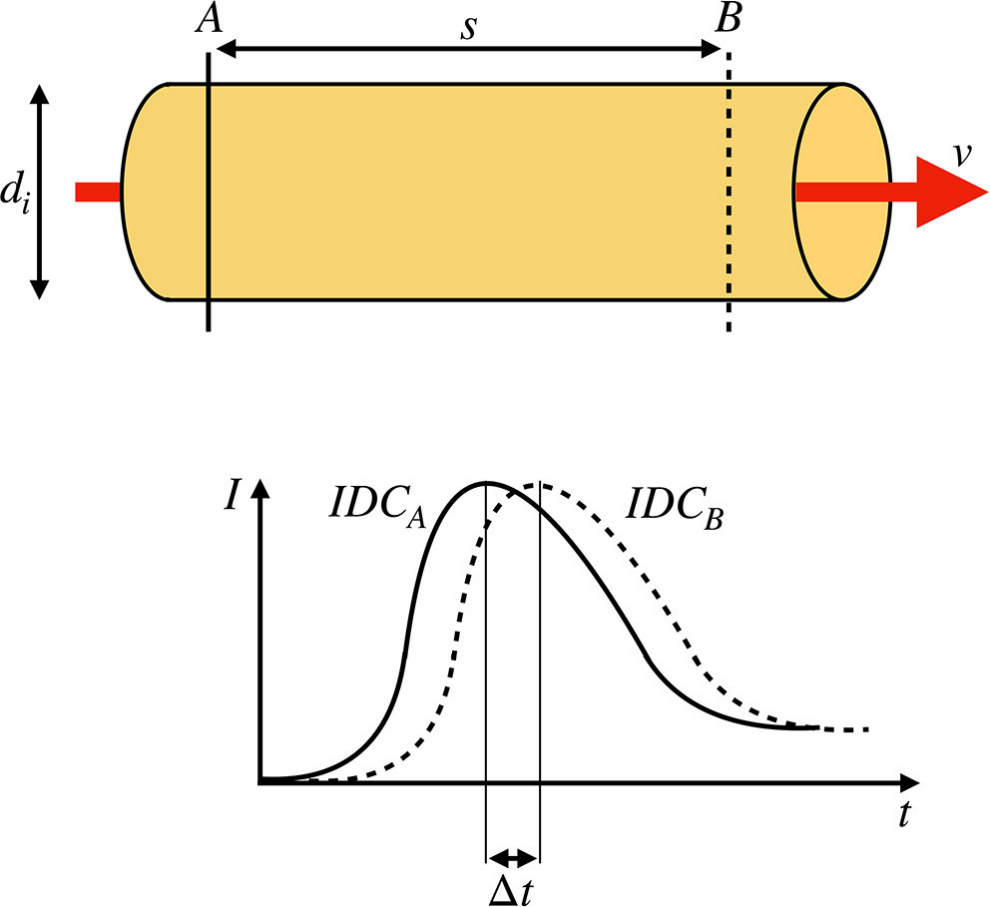
**(Top)** Sketch of a rigid pipe with an inner diameter of *d*_*i*_ containing a fluid flow with a constant mean flow velocity *v*. Two measurement points *A* and *B* with a distance of *s* are defined. **(Bottom)** Two indicator dilution curves (IDCs) obtained at the locations *A* and *B* by optical measurement. The transit time Δ*t* is defined as the shift of the curves as indicated by the shift of the maxima.

In the last two decades, optic-based medical systems were introduced to intraoperatively visualize vascular structures via fluorescence angiography (Raabe et al., 2003; Kamp et al., 2012). Fluorescence angiography provides valuable spatial and temporal information to the surgeon regarding a vessel's patency or an area's perfusion. The vascular function is essential to the human brain due to the brain's low hypoxia tolerance (Silbernagl and Despopoulos, 2003). The electroencephalography (EEG) shows a ceased oscillation within seconds after cardiac arrest (Choi, 1990). Apoptosis and cognitive impairment are the inevitable results of insufficient cerebral perfusion (Pulera et al., 1998; Ortapamuk and Naldoken, 2006). Therefore, a rapid response is needed in the operating theater where revascularization should restore the vascular function (Lawton and Lang, 2019). However, a subjective inspection of the patency by the surgeon may not be sufficient for intraoperative decision making. Extracranial to intracranial bypass surgery has a mortality of 10.2% within 1 month of the index date and a 5–9% recurrence rate after surgery. These high rates can be traced back to inadequate blood flow through the donor vessel (Chen et al., 2018). Therefore, a quantitative approach for intraoperative blood volume flow assessment is needed to check the quality of the procedure (Kamp et al., 2012). A current clinical routine for blood volume flow assessment involves the usage of an ultrasonic flow probe. The accuracy of the latest state-of-the-art clinical flow probe is ±10% (Transonic, 2019). Increased operation time, equipment, and an interruption of the surgical workflow by changing the surgeon's instrument are downsides of this method. Moreover, the added mechanical stress can compromise the vessel's integrity and thus poses a risk to the patient (Amin-Hanjani et al., 2006). Additionally, narrow and deep working channels are a challenge for the cumbersome probes. Hence, a method without tissue contact would be advantageous. Intraoperative fluorescence angiography is a non-invasive method and can be used in combination with an intravenously injected indicator to observe the blood's dynamic (Raabe et al., 2003; Weichelt et al., 2013). In general, two approaches are used to calculate the volume flow. The first approach is a one-point measurement where the temporal intensity signal in one point or ROI (mean value of a region of interest) is analyzed. The flow is then calculated following the fundamentals of the indicator dilution theory (Saito et al., 2018). However, one drawback of this approach is the dependency of the indicator dilution theory on absolute measured concentrations that are not given in fluorescence angiography. Concentrations cannot be simply calculated from the backscattered fluorescence signals without solving an ill-posed inverse problem. Additionally, no constancy of mass is given since the injected bolus does not necessarily traverse the vessel, which is measured due to the complex cerebrovascular branching. The second approach is a two-point measurement. The blood volume flow $\dot{V}$ is calculated as shown in Equation (1), where *A* is the vessel's cross-sectional area, $\bar{v}$ the mean blood flow velocity, *d*_*i*_ the inner diameter of the vessel, and Δ*t* is the transit time of the indicator bolus to travel the distance *s* (Weichelt et al., 2013). We base our method on the second approach since its preconditions are promising. The determination of *d*_*i*_ and *s* is done by machine vision and will not be discussed in this paper. To calculate Δ*t*, two indicator dilution curves (IDCs) are obtained by monitoring the spatiotemporal propagation of the indicator bolus at two distinct locations along the vessel of interest (see Figure 1). The transit time of the bolus can be extracted as the temporal shift of the two IDCs.

(1) $$\begin{array}{l} \dot{V}=A\cdot \bar{v}=\frac{\pi \cdot d_{i}^{2}\cdot s}{4\cdot \Delta t} \end{array}$$

The accuracy of the calculation of $\dot{V}$ strongly depends on the errors in measuring *s*, *d*_*i*_, and Δ*t*. The quantification of these errors and their error propagation is necessary and not sufficiently investigated yet (Cimalla et al., 2008; Weichelt et al., 2013). The accuracy in the measurement of the distance *s* (geodesic length of the vessel's centerline) is of a magnitude of 3% but shall not be discussed in this paper (Naber et al., 2020a).

In this paper, we focus on evaluating the performance of methods ascertaining the value of Δ*t* and its quantitative statistical error. Please note that this research paper only investigates the statistical error in determining the transit time (or delay of a signal). The systemic error of the optical transit time measurement in fluorescence angiography is not the focus of this paper but is part of our research (Naber et al., 2020b). The systemic error will be shortly explained in the following paragraph.

Equation (1) is derived from the general definition of volume flow $\dot{V}=\int v\left(r,\phi \right)dA$ with the assumption that is the mean value of *v* over *A*. The measured flow velocity via fluorescence angiography is not the mean value of *v*(*r*, ϕ) due to the limited penetration depth of exciting and fluorescent photons. Flow velocities that are close to the surface and closer toward the direction of incidental excitation light are more likely to be observed than photons in greater depth. Therefore, we propose to introduce a spatial weighting factor *w*(*r*, ϕ) to calculate the volume flow $\dot{V}=\int v\left(r,\phi \right)\cdot w\left(r,\phi \right)dA$. The normalization of the absolute flow velocity field *v*(*r*, ϕ) to $\bar{v}$ is convenient since $\bar{v}$ can be set before the integral. Discretizing the integral results in $\dot{V}=A\cdot \bar{v}\cdot \sum v_{relative}\left(r,\phi \right)\cdot w\left(r,\phi \right)$. During laminar flow, *v*_*relative*_ is of parabolic shape and *w*(*r*, ϕ) can be obtained by simulating the photon's propagation in turbid fluorescent media (see Naber et al., 2020b). The term in front of the sum ($A\cdot \bar{v}$) is the calculated flow in Equation (1). Consequentially, the remaining sum represents the systemic error mentioned earlier.

Data sets containing two corresponding indicator dilution curves with a ground truth of the transit time Δ*t* are needed to investigate the statistical error of Δ*t*. Although *in vivo* IDCs are monitored frequently in clinical routines and are thus widely available, they lack a sufficient and trustworthy ground truth of the transit time (Reuter et al., 2010). Additionally, they contain temporally correlated and uncorrelated noise that cannot be separated from the desired signal [described by the additive noise model *m*(*t*) = *s*(*t*) + *n*(*t*) with *m*(*t*) as measured, *s*(*t*) as desired signal and *n*(*t*) as noise]. Therefore, making the analysis of the influence of the noise is impossible. The state-of-the-art surgical microscopes have a maximum sampling frequency of *f*_*sampling*_ = 60 *Hz*, which is sufficient to discretize the continuous IDC (Nyquist Shannon sampling theorem). Simply shifting an *in vivo* IDC would result in identical samples having identical noise, which is unintended. Artificially creating sub-samples (reasonable samples in between the measured ones) would be a simple method to increase the number of samples and allow shifting of one IDC. To obtain uncorrelated sub-samples a mathematical model is used, which interpolates the signal. The model applies bias to the performance of methods ascertaining the time shift which is undesirable. Consequentially, a credible performance evaluation of methods ascertaining the transit time Δt based on *in vivo* IDCs cannot be assured by merely duplicating and delaying an IDC for these three main reasons.

Therefore, we propose a method to compute highly adaptable data sets of two corresponding and differently sub-sampled IDCs with a ground truth of the transit time using an *in silico* model. Even though an *in vitro* flow phantom could provide a similar data set, *in silico* generated data sets are low cost, accurate, and easily configurable to the desired field of application (Kung et al., 2011). Subsequently, these data sets will be used to evaluate and enhance current methods ascertaining the transit time Δ*t*, thereby increasing its temporal accuracy that is not yet suitable for clinical studies (Cimalla et al., 2008; Weichelt et al., 2013). Achieving a higher temporal accuracy by increasing the sampling rate of the measuring device is possible. In a noise-free signal, the maximum error of an ideal measurement is equal to half of the quantization interval, in our case, equal to $\frac{1}{2\cdot f_{sampling}}$. In a noisy signal, the accuracy will decrease drastically with a decreasing signal-to-noise ratio (SNR). Furthermore, an increase in the sampling rate is limited since the noise level is often coupled reciprocally to the integration time. Therefore, we propose to fit mathematical models to the data points to interpolate the signal. This can enhance the accuracy in the temporal detection of events (Ellis et al., 2015). The proposed models are commonly used in the IDC-based measurement of blood volume and cardiac output to suppress noise, artifacts, and increase the accuracy of the measurement (Hamilton et al., 1932; Meier and Zierler, 1954; Zierler, 2000; Mischi et al., 2003, 2008).

Two hypotheses were set up as follows:

1. Using mathematical models to re-continualize the dilution curves, there is a decrease in the error in transit time measurements compared to using the raw data.
2. A sub-frame rate accuracy can be accomplished by combining a suitable configuration of a mathematical model, extracted features, and pre-processing of raw data.

## 2. Methods

The overarching goal is to correctly obtain the indicator bolus transit time. The accuracy is limited by the frame rate and a sub-frame rate accuracy is required. This can be solved by fitting an appropriate analytical function to the measured curves and obtain the time delay of two IDCs from the time delay of both fits. Methods to determine the most appropriate analytical function are described in section 2.2. The missing ground truth is a major problem to properly perform the proposed investigation. Therefore, IDCs are simulated, shifted by a defined time as the ground truth for the bolus transit time, and superposed with Gaussian noise. The methodology for the generation of the data set containing the ground truth is described in section 2.1. We are not seeking to perfectly model *in vivo* IDCs but rather to compute similar curves. Multiple assumptions are set. First, the bolus transit time equals the mean blood volume transit time. Second, the real fluorescence intensity curve equals the simulated indicator bolus curve. Finally, the simulated indicator bolus curve equals the realistic indicator solution curve in a cerebral vessel.

All computation is done on a computer with an Intel i7-6500U processor.

### 2.1. IDC Generation

The following requirements were set for the data sets of two corresponding IDCs:

- The computed curves should mimic indicator dilution curves.
- The curves' morphology should be highly configurable.
- The ground truth transit time of the two curves in a data set must be known.
- The samples of the curves should be shifted relative to each other (so using two curves with not identical samples ensuring the depiction of two curves taken at two different locations).
- Different noise levels should be applicable.

The finite element-based software COMSOL Multiphysics (version 5.4) with the packages Computational Fluid Dynamics (laminar flow) and Chemical Reaction Engineering (transport of concentrated species and transport of diluted species) was used for this *in silico* model. The *.mph* file to reproduce the results is presented in the Supplementary Materials. The calculation of the motion of the fluid is based on the Navier–Stokes equations and the continuity equation (see Equations 2 and 3 with **u** as the fluid velocity, *p* the pressure, ρ the density, μ the dynamic viscosity, and **F** external forces). The nabla operator in Equations (2) to (6) indicates the coverage of all spatial dimensions (which is comparable to the analytical solution of the convection-diffusion of a liquid by Taylor–Aris; Aris, 1956).

(2) $$\begin{array}{l} \rho \left(\frac{\partial \text{u}}{\partial t}+\text{u}\cdot \nabla \text{u}\right)=-\nabla p+\nabla \cdot \left(\mu \left(\nabla \text{u}+{\left(\nabla \text{u}\right)}^{T}\right)-\frac{2}{3}\mu \left(\nabla \cdot \text{u}\right)\text{I}\right)+\text{F} \end{array}$$

(3) $$\begin{array}{l} \frac{\partial \rho }{\partial t}+\nabla \cdot \left(\rho \text{u}\right)=0 \end{array}$$

Assuming laminar flow, an incompressible fluid and no external forces (such as gravity) the continuity equation yields

(4) $$\begin{array}{l} \nabla \cdot \text{u}=0, \end{array}$$

the term $-\frac{2}{3}\mu \left(\nabla \cdot \text{u}\right)\text{I}=0$ and **F** = 0.

The dye will spatially diffuse due to its concentration gradient. Hence, the direction of the diffusive flux can be normal to the fluid flow direction. This is expressed by the convection-diffusion equation (Equation 5 with *c* being species concentration, **J**_**Diff**_ being the diffusive flux and **R** describing sources or sinks of *c*). The diffusive flux can be approximated by Fick's first law (Equation 6 with *D* being the diffusion coefficient). Since no sources or sinks are present, **R** = 0.

(5) $$\begin{array}{l} \frac{\partial c}{\partial t}+\nabla \cdot {\text{J}}_{\text{Diff}}+\text{u}\cdot \nabla c=\text{R} \end{array}$$

(6) $$\begin{array}{l} {\text{J}}_{\text{Diff}}=-D\nabla c \end{array}$$

The equations are fully coupled and solved by the PARDISO (PARallel DIrect SOlver) solver which is based on the LU decomposition (Lower-Upper). The relative tolerance in the calculation of each measuring point is set to 0.1%.

The modeling in this paper is performed using COMSOL Multiphysics as a tool for numerical simulation. Alternatively, other software can be used analogously (Ansys, OpenFOAM, or similar). Analytical approaches can be used as well for this investigation such as the Taylor–Aris approach. The numerical approach via COMSOL enables the implementation and calculation of more complex geometries and the ability to calculate spatially resolved concentrations. This is advantageous for future applications. In this paper, the used geometry and properties are simplified and not realistic representatives of the circulatory system and therefore merits of the methods used for computing are irrelevant.

**Figure 2:**
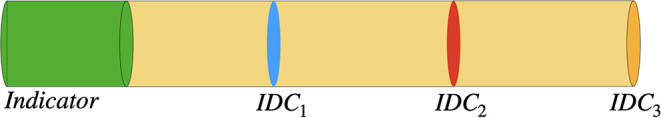
Sketch of the rotationally symmetric geometry with a section containing a homogeneously distributed indicator (green area) and a blood analog (yellow area). After applying a laminar flow, concentration curves are obtained as the mean concentration at the pipe's cross-sectional area at the three marked locations (blue, red, and orange). The corresponding dilution curves are shown in Figure 3. Please note that the scale of the figure does not represent the scale used in the *in silico* model.

The fluid was modeled as a homogeneous liquid. In contrast, blood is a cell suspension containing the liquid plasma and the solid cells. Since the indicator of interest (Indocyanine Green—ICG) binds primarily to the proteins in the blood plasma, it will be affected by a heterogeneous distribution of cells (Alander et al., 2012). To reduce the computational effort in our model, this heterogeneous distribution was not taken into account and simplified to a homogeneous distribution. A rotationally symmetric geometry was defined. It is divided into a section containing blood analog (water with glycerol and protein powder) and a section containing a homogeneous concentration of the indicator (ICG) dissolved in the blood analog (see Figure 2, the used geometry mimics an in-house flow phantom. The geometry, COMSOL settings and the defined parameters are attached in the Supplementary Material, section 1.1). Consequentially, the indicator concentration is a rectangular function at *t* = 0 and mimics an abrupt injection. Finally, multiple lateral locations were defined where the mean indicator concentration across the cross-sectional area was measured. Thereby, different morphologies were computed depicting increasing degrees of dilution. We used a distance of 50 cm between the three different measurement locations. The distance from the heart to the brain is ~50 cm, which would mimic an injection via a central venous catheter and the other distances peripheral (e.g., arm of the patient) injections (Arieli and Marmur, 2017). The following input parameters, properties, and boundary conditions were defined:

- Inner radius: *r*_*i*_ = 2 *mm*
- Outer radius: *r*_*o*_ = *r*_*i*_
- Rigid vessel wall: *E* = 0
- Applied volume flow: $\dot{V}=150\frac{ml}{min}$
- Inflow modeled as a ramp function reaching the designated volume flow in 1 s.
- Open outlet with: *p* = 1 *atm*
- Laminar fluid flow and convection (*Re*- Reynolds number): *Re* ≈ 600 < *Re*_*critical*_
- Incompressible fluid: ρ = *constant*
- Non-Newtonian fluid behavior modeled by the Casson model (see Supplementary Material, section 1.1)
- No wall slip: *u*(*r* = *r*_*i*_) = 0
- No external pressure or hydrostatic pressure
- No gravitational force.

The volume flow is chosen based on reports on physiological values of cerebral arteries of this size. Thereby, a linear regression of the weighted average (weighted on the sample size of each report) is performed and the value for the chosen diameter is rounded to the closest multiple of $25\frac{ml}{min}$ (Nakayama et al., 2001; Chen et al., 2004; Mujagic, 2013; MacDonald and Frayne, 2015; Zarrinkoob et al., 2015). The modeled volume flow is continuous and not pulsatile. This simplification can be assumed as valid in distal arteries. It was shown that the Gosling pulsatility index is significantly reduced from proximal to distal measurements of volume flow in cerebral arteries (Gosling and King, 1974; Zarrinkoob et al., 2016).

**Figure 3:**
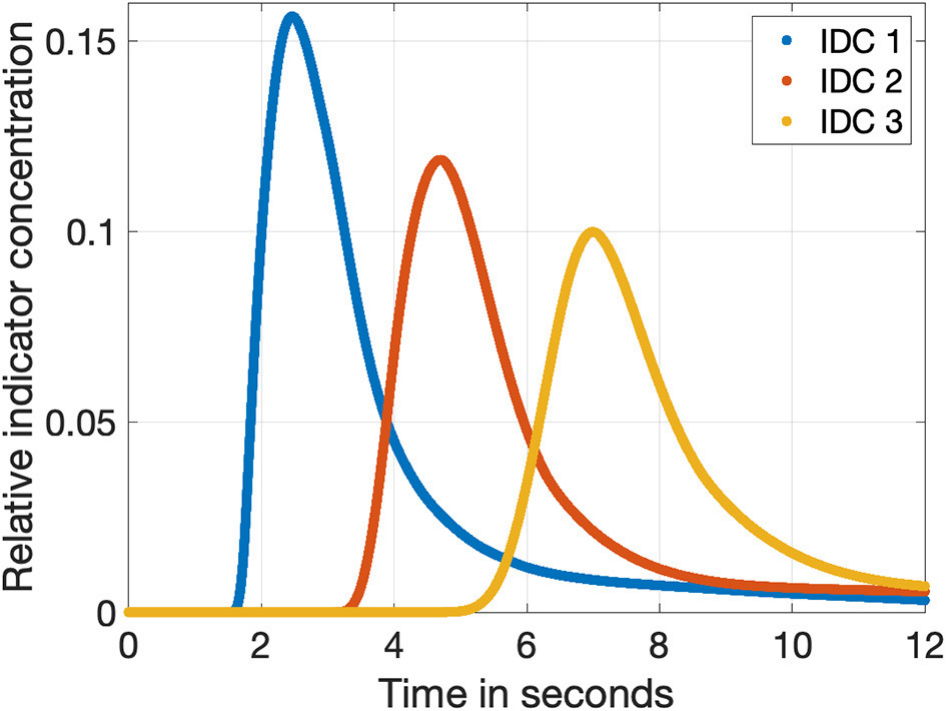
The three indicator dilution curves obtained from the fluid flow model with a sampling rate of 1,200 Hz. The relative concentration is normalized to the input concentration *c*_*ICG*_. The blue, red, and orange curves show the dilution curves in a distance of 50, 100, and 150 *cm* to the injection side as shown in Figure 2, respectively.

For the simulation of the flow and the evaluation of the mean indicator concentration, a mesh with 175,176 elements was built. Each mesh element consisted of triangles with a radially decreasing side length ranging from 10^−4^ to 10^−6^ *m*. To obtain the three IDCs with different morphologies, the time frame of the simulation was set from 0 to 12 *s* with a sampling rate of *f*_*sampling*_ = 1, 200 *Hz*. Therefore, each computed data set consisted of 1, 200 · 12 = 14, 400 samples. The sampling frequency is related to the frame rate of common surgical microscopes of 25 and 60 *Hz*. As a result of this sampling process with 1, 200 *Hz*, we obtained $\frac{1,200}{25}=48$ different sub-samples at 25 *Hz* and $\frac{1,200}{60}=20$ different sub-samples at 60 *Hz*. This held the advantage that it also increases the variability in shifting the curve. The raw IDCs are shown in Figure 3. After computing the raw IDCs, the data sets were imported into MathWorks MATLAB R2019b.

**Figure 4:**
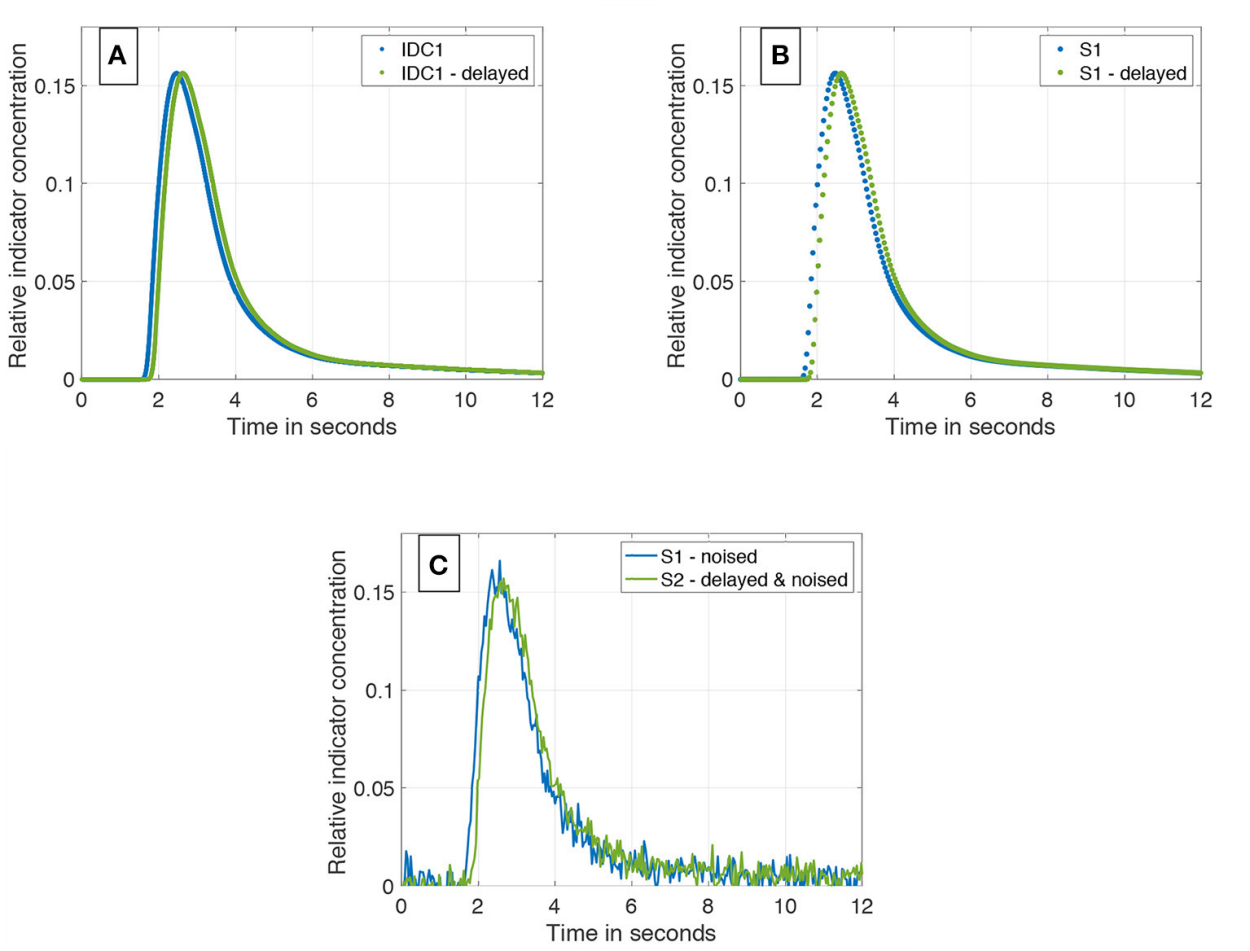
**(A)** IDC1 in blue without any manipulations. In green, the same curve delayed by a transit time Δ*t* = 160 *ms*. Both are sampled with 1,200 *Hz*. **(B)** Sub-sampling of the two curves from **(A)**. Both curves are sub-sampled to 25 *Hz*. **(C)** Final data set containing two differently sub-sampled curves; one of them temporally shifted by the ground truth transit time Δ*t*. White Gaussian noise is applied to both IDCs (*SNR* = 20 *dB*).

The following steps were applied to fulfill the requirements:

- Duplication of one IDC (see Figure 4A).
- Temporal shift of one IDC by a known value (ground truth) ranging from 1 to 4 frames in the case of 25 *fps* (2 frames to eight frames in case of 60 *fps*; see Figure 4A).
- Different sub-sampling to the desired sampling rate (e.g., 25 or 60 *fps*; see Figure 4B).
- Application of white Gaussian noise to both curves to represent Johnson–Nyquist noise (SNR ranges from 8 to 20 *dB*; see Figure 4C).

The motivation of duplicating one IDC is strongly linked to the expected temporal shift. The field of view in cerebrovascular surgery is ~3 × 3 *cm*^2^, the mean flow velocity is about ~$10-50\;\frac{cm}{s}$ and a typical exposed length of a vessel is 1–2 cm (Cieslicki and Ciesla, 2005; Ebner et al., 2011; MacDonald and Frayne, 2015; Zarrinkoob et al., 2015). The expected morphological changes of the IDCs is negligible; in the Supplementary Material (section 3.6), the magnitude of change is shown. Therefore, the computed IDCs are duplicated and shifted by the proposed range, which relies on the expected clinical circumstances.

Different sources of noise will appear in measurements, e.g., thermal noise from the recording device and patient-related noise such as the heart rate. We chose to represent the Johnson–Nyquist noise in our data set by additive white Gaussian noise due to its similar characteristic. Additive white Gaussian noise is described by the probability density function shown in Equation (7) with μ as the mean value and σ as the standard deviation.

(7) $$\begin{array}{l} f\left(n\right)=\frac{1}{\sqrt{2\pi \sigma^{2}}}e^{-\frac{{\left(n-\mu \right)}^{2}}{2\sigma^{2}}} \end{array}$$

μ was set to zero and σ is calculated using the definition of the SNR (Equation 8).

(8) $$\begin{array}{l} SNR=10\cdot log_{10}\left(\frac{P_{Signal}}{P_{Noise}}\right)\;dB \end{array}$$

With the correlation

(9) $$\begin{array}{l} P_{Noise}=2\sigma^{2} \end{array}$$

of the noise power *P*_*Noise*_, the standard deviation σ and the approximated signal power

(10) $$\begin{array}{l} P_{Signal}=\sum |s\left(t\right){|}^{2} \end{array}$$

of the noise-free IDC samples *s*(*t*), the standard deviation σ can be calculated as

(11) $$\begin{array}{l} \sigma =\sqrt{\frac{\sum |s\left(t\right){|}^{2}}{2\cdot 10^{\frac{SNR}{10\cdot dB}}}} \end{array}$$

We did not add any other noise due to the lack of information on its characteristic since mostly the separation of desired signal and noise is not possible. In addition to Johnson–Nyquist noise, we could consider to include shot noise. The quantum fluctuation of the photons hitting the detector are dependent on the scattering, absorption, and fluorescence events in a tissue slab, which are stochastically determined processes. Hence, they can be interpreted as shot noise. Following the central limit theorem, the uncertainties of the interaction of a photon relies on the superposition of multiple randomized events and therefore shot noise can be assumed as normally distributed and modeled by additive white Gaussian noise (Wohland et al., 2001). Other influences of noise are excluded in this investigation. Additive white Gaussian noise tests the robustness of the proposed mathematical models against disturbance. This is rather in focus of the investigation than the ideal realistic representation of all sources of noise.

The application of noise is done for each data set independently, so each time a new noise set is generated and added to the signal. The application of noise on the signal assumes that the error in transit time measurement is linked to amplitude noise and is therefore an appropriate representation of disturbing influences on the measurement.

The pipeline is shown in Figures 4A–C.

In summary, different raw indicator dilution curves were simulated in COMSOL Multiphysics and imported to MathWorks MATLAB to compute different sub-samples, applying a temporal shift and noise. In total, 12,096 data sets were computed:

- 3 raw IDCs with different morphology;
- 2 sampling rates: *f*_*sampling*, 1_ = 25 *fps* & *f*_*sampling*, 2_ = 60 *fps*;
- 12 different sub-sampling combinations;
- 7 different SNR levels (8–20 dB in 2 *dB* steps);
- 6 independent realizations for each noise level;
- 4 different temporal shifts (transit times).

As a result, a large amount of diverse data sets, each containing two corresponding IDCs [*c*_1_(*t*) and *c*_2_(*t*)] with a known ground truth transit time, are given and ready for the following evaluation of different methods ascertaining the transit time Δ*t*. In the case that methods are applied to both curves, only a *c*(*t*) is used for better intelligibility.

### 2.2. Evaluation of Methods Ascertaining the Transit Time Δ*t*

MathWorks MATLAB R2019b was used for the following evaluation and analysis.

Typical analysis of transit time is based on feature-based methods such as peak to peak distances or using the cross-correlation of both signals as a tool to determine the transit time (Cimalla et al., 2008). Using artificial up-sampling, these methods are able to compute sub-frame rate transit times. However, artificial up-sampling does not add information to the data and can provoke artifacts that manipulate the analysis in a negative manner.

We propose to fit a mathematical model to the data sets of both IDCs *c*_1_(*t*) and *c*_2_(*t*) and then compute the transit time as the shift of those two mathematical functions. This paper takes four models into consideration: A parabola function, the local density random walk function (LDRW), the gamma variate function, and the mono-exponential function. The parabola function is chosen due to its simplicity and robustness of fitting; it takes only the data points around the peak into account. The LDRW function is based on the theory of diffusion of the indicator by drift models. It regards the indicators movement as a longitudinal diffusion superimposed on a linear convection and is valid for an instantaneous injection, which fits our problem (Wise, 1966; Norwich and Zelin, 1970). The gamma variate function is derived from modeling a unidirectional constant flow as a series of multiple mixings in sequential compartments of the same volume with only one input and output, which fits our problem as well (Schlossmacher et al., 1967; Davenport, 1983). Both the LDRW and gamma variate function take the whole curve into account. However, both are not able to represent recirculation. The mono-exponential function describes the washout of the indicator and only takes the descending arm of the curve into account. It was developed to extrapolate the dilution curves on the descending arm to estimate the curves course without recirculation (Hamilton et al., 1932).

Different features will be used to extract the transit time Δ*t* from two fitted mathematical functions. Each feature is function specific and will be described in detail in the following section.

#### 2.2.1. Cutting the Data Set

Before fitting mathematical models to the two curves of a data set, a pre-processing step is needed. Each IDC needs to be cut to ensure a robust fitting. Two examples are as follows: First, prior to the arrival of the indicator zeros are recorded. Including them into the fitting algorithm does not make any sense and can cause problems. Second, fitting the parabola model to the whole signal does not make sense either, therefore the peak is detected and only data points surrounding it are considered for fitting.

Addressing the problem of the first example, the data points prior to the appearance of the indicator should be removed since they contain no information and are basically zeros with noise applied to it. Equation (12) has shown to be empirically appropriate to find this cut point *t*_*cut*1_ on the abscissa representing the appearance of the indicator despite of the impact of noise before the peak of the IDC (*c*_*max*_ is the maximum concentration, *t*_*up*_ only considers the data points left of the peak, and *t*_*down*_ only considers the data points right of the peak). To determine the elements of the equation, a moving average with a window length of 20 samples is used to reduce the impact of noise on the signal. To ensure unambiguity, the search for the elements on the up slope is performed from left to right and on the down slope from right to left. This pre-processing is applied on all data sets independently to the further processing.

(12) $$\begin{array}{l} t_{cut1}=t_{up}\left(0.2\cdot c_{max}\right)-0.1\cdot \left[t_{down}\left(0.5\cdot c_{max}\right)-t_{up}\left(0.5\cdot c_{max}\right)\right] \end{array}$$

**Figure 5:**
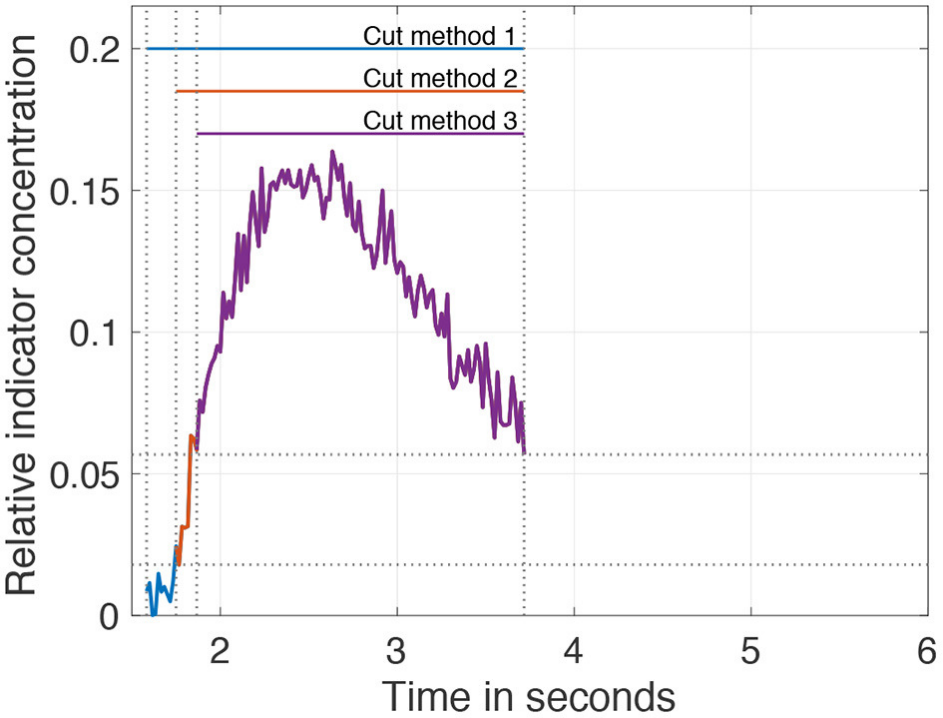
Visualization of the three different pre-processing (cutting) methods on the data. The cut level is set to 40% of the maximum. After applying cut method 3, the purple curve remains. Applying cut method 2 results in the curve consisting of purple and red. Application of cut method 1 results in the curve of all three colors purple, red, and blue.

Addressing the problem of the second example, it is reported that cutting the data as a pre-processing step enhances the robustness and accuracy of the fit. Seven different levels of cutting are introduced: they range from *t*(0.2 · *c*_*max*_) to *t*(0.8 · *c*_*max*_) in steps of 0.1. Further, it is reported that an unequal cutting to the left compared to the right of the peak is favorable due to different information content in the data (e.g., exclusion of recirculation; Borges et al., 2012). For this purpose, 3 cutting methods are additionally introduced and depicted in Figure 5.

1. No further cutting left of the peak and variable cutting levels right of the peak.
2. Cutting left of the peak at half the value of the variable cutting to the right of the peak.
3. Variable but identical cutting left and right of the peak.

In summary, 21 different variations (7 cut levels times 3 cut methods) to cut the IDC as a pre-processing step are possible and will be evaluated in this analysis.

The mathematical models are described in the following sections as well as the initial estimation of their parameters *p*_*init*_ = (*p*_1,*init*_, *p*_2,*init*_, …, *p*_*n, init*_). The initial estimation is needed to run a least squares optimization algorithm to determine the function's parameters *p* which fit the data best. Since indexed variables are easier to handle the input data *c*(*t*) is described as the set of data points [*t*(*k*), *c*(*k*)] with ∀*k* ∈ [1, *K*] (*K* being the number of data points). Therefore, we solve the problem

(13) $$\begin{array}{l} \min_{p}\left\{F_{fit}\left(p\right)\right\}=\min_{p|p_{i}\in \left[p_{lb},p_{ub}\right]}\left\{\sum_{k=1}^{K}|f_{model}\left(p,t\left[k\right]\right)-c\left[k\right]{|}^{2}\right\} \end{array}$$

with *p*_*lb*_ being the lower bound and *p*_*ub*_ the upper bound of the respective parameters *p*_*i*_. To solve Equation (13), different algorithms like the “trust-region-reflective” or the “Levenberg–Marquardt” algorithms are suitable. The “trust-region-reflective” algorithm is used in our study (More and Sorensen, 1983).

The time shift of the functions can be determined after fitting the mathematical models to the pre-processed (cut) input data (two IDCs). The features used to determine the time shift are specific to the mathematical models and described in the corresponding following sections.

#### 2.2.2. Parabola Model

The parabola model is defined as shown in Equation (14). This model is fitted to the data points around the peak (according to the cutting method specified in section 2.2.1) of the IDC.

(14) $$\begin{array}{l} f_{Parabola}\left(p,t\right)=-p_{1}\cdot {\left(t-p_{2}\right)}^{2}+p_{3} \end{array}$$

The parabola has a distinct morphology that facilitates setting up *p*. Therefore, the input *c*(*t*) was smoothed by a sliding rectangular window with a window size of 20 samples and *p* determined as shown in the following equations:

(15) $$\begin{array}{l} p_{1,init}=\frac{c\left(t_{n}\right)-c_{max}}{{\left(t_{n}-t\left(c_{max}\right)\right)}^{2}} \end{array}$$

(16) $$\begin{array}{l} p_{2,init}=t\left(c_{max}\right) \end{array}$$

(17) $$\begin{array}{l} p_{3,init}=c_{max} \end{array}$$

No boundaries are needed for the optimization of the parameters *p* of the parabola.

To determine the shift of the two fitted parabolas of one data set, the maximum of each parabola is used as feature.

#### 2.2.3. Local Density Random Walk Model

The LDRW model function is defined as shown in Equation (18). This model can be fitted to the whole IDC.

(18) $$\begin{array}{l} f_{LDRW}\left(p,t\right)=p_{1}\cdot \left(\frac{e^{p_{2}}}{p_{3}}\right)\cdot \sqrt{\frac{p_{2}\cdot p_{3}}{2\pi \left(t-p_{4}\right)}}\cdot e^{-\frac{p_{2}}{2}\cdot \left(\frac{p_{3}}{t-p_{4}}+\frac{t-p_{4}}{p_{3}}\right)} \end{array}$$

According to the approach of Mischi et al. (2003) and Bogaard et al. (1984) the parameters are calculated as shown in the following equations. *p*_1,*init*_ represents the total amount of injected dye. Consequentially, it can be expressed as the integral of *c*(*t*) or in the discrete case as a sum of the products (Equation 19). *p*_2,*init*_ represents the slope of the ascending arm. *p*_3,*init*_ represent the abscissa coordinate of the maximum. *p*_4,*init*_ represents the zero time of the distribution and in our case it is slightly shifted away from the distributions maximum to be sure to not prune the distribution at the beginning because our function is defined only for positive values of *t* − *p*_4_ and therefore optimization will be only performed for *t* > *p*_4_.

(19) $$\begin{array}{l} p_{1,init}=\frac{1}{2}\cdot \overset{N}{\underset{i=0}{\sum }}\left(t_{i+1}-t_{i}\right)\cdot \left[c\left(t_{i}\right)+c\left(t_{i+1}\right)\right] \end{array}$$

(20) $$\begin{array}{l} p_{2,init}=\frac{1}{2}\cdot \frac{\left|t\left(c_{max}\right)-t_{0}\right|}{c_{max}} \end{array}$$

(21) $$\begin{array}{l} p_{3,init}=\frac{1}{2}\cdot \left(t_{n}-t_{0}\right) \end{array}$$

(22) $$\begin{array}{l} p_{4,init}=t_{0}-\frac{1}{5}\cdot |t\left(c_{max}\right)-t_{0}| \end{array}$$

The boundaries for *p* were set as $\left(p_{1},p_{2},p_{3},p_{4}\right)\in \left(\left[0,\infty \right),\left[0,\infty \right),\left[\frac{p_{3,init}}{100},\infty \right),\left[0,t_{0}\right]\right)$.

To determine the shift of two fitted LDRW functions, the maximum as well as the maximum and minimum of the first and second derivative of each LDRW function are used as features. Further, the cross-correlation of the two LDRW functions is used to determine the time shift. It is applied to the two LDRW functions and their first derivative, as well as to the functions and their first and second derivative with an additional linear temporal interpolation with a factor of 100 (the functions are continuous but MATLAB is a vector and matrix based software and the calculation of the cross-correlation requires a discrete input and therefore the functions are sampled with a sampling rate 100 times higher than the input sampling rate for the fitting, e.g., *f*_*sampling*_ = 25 *Hz*, then the input for the calculation had an *f*_*sampling*_ = 2, 500 *Hz*. We assume this accuracy with maximum error of $\frac{1}{50}frame$ is sufficient).

#### 2.2.4. Gamma Variate Model

The gamma variate model is defined as shown in Equation (23). This model can be fitted to the whole IDC.

(23) $$\begin{array}{l} f_{Gamma}\left(p,t\right)=p_{1}\cdot {\left(t-p_{4}\right)}^{p_{2}}\cdot e^{-\frac{t-p_{4}}{p_{3}}} \end{array}$$

According to the approach of Madsen (1992) and Mischi et al. (2008) the parameters are calculated as shown in the following equations. *p*_1_ and *p*_2_ represent the coordinates of the maximum (obtained by differentiating the function and setting it equal to zero). *p*_3_ represents the slope of the decreasing arm of the function. *p*_4_ represents the zero time of the distribution and in our case it is slightly shifted away from the distribution's maximum to avoid syntax errors.

(24) $$\begin{array}{l} p_{1,init}=\frac{e^{p_{2,init}}\cdot c_{max}}{{\left(p_{2,init}\cdot p_{3,init}\right)}^{p_{2,init}}} \end{array}$$

(25) $$\begin{array}{l} p_{2,init}=\frac{t\left(c_{max}\right)-p_{4,init}}{p_{3,init}} \end{array}$$

To calculate *p*_3,*init*_, an additional step is needed. Therefore, a moving average with a window size of 20 samples is applied and the endpoint [*t*_*SW, n*_, *c*(*t*_*SW, n*_)] is used to calculate *p*_3,*init*_.

(26) $$\begin{array}{l} p_{3,init}=-\frac{t_{SW,n}-t\left(c_{max}\right)}{ln\left(c\left(t_{SW,n}\right)\right)-ln\left(c_{max}\right)} \end{array}$$

(27) $$\begin{array}{l} p_{4,init}=t_{0}-\frac{1}{5}\cdot |t\left(c_{max}\right)-t_{0}| \end{array}$$

The boundaries for *p* were set as $\left(p_{1},p_{2},p_{3},p_{4}\right)\in \left(\left[0,\infty \right),\left[0,\infty \right),\left[\frac{p_{3,init}}{100},\infty \right),\left[0,t_{0}\right]\right)$.

To determine the shift of two gamma variate functions, the same features and methods are used as for the LDRW function.

#### 2.2.5. Mono-Exponential Model

The mono-exponential model is defined as shown in Equation (28). This model is fitted to the descending arm of the data set. Thereby, the descending arm is defined as the data points *t* > *t*(*c*_*max*_) after running a moving average on *c*(*t*) with a window size of 20 samples.

(28) $$\begin{array}{l} f_{MonoEx}\left(p,t\right)=p_{1}\cdot e^{p_{2}\cdot \left(t-p_{3}\right)} \end{array}$$

According to Brands et al. (2011) the parameters are calculated as shown in the following equations. To determine *p*_1,*init*_ and *p*_3,*init*_, the smoothed data are used. *p*_1,*init*_ and *p*_3,*init*_ represent the coordinates of the maximum of the function, hence the start of the defined sector of the mono-exponential function. *p*_2,*init*_ represents the slope of the function.

(29) $$\begin{array}{l} p_{1,init}=c_{max} \end{array}$$

(30) $$\begin{array}{l} p_{2,init}=\frac{1}{t_{n}-p_{3,init}}\cdot ln\left(\frac{c\left(t_{n}\right)}{p_{1,init}}\right) \end{array}$$

(31) $$\begin{array}{l} p_{3,init}=t\left(c_{max}\right) \end{array}$$

No boundaries are needed for the parameter optimization *p* of the mono-exponential model.

Determining the shift of two Mono-Exponential functions is not trivial since their maxima and minima depend solely on the sections of *c*(*t*) to which they are fitted to. So, no maximum or minimum of the function can be used. Therefore, a feature is introduced that describes the Euclidean distance of the normalized function to a defined point. Both axes are normalized, *c*(*t*) to *c*_*max*_ and *t* to *t*_*n*_ − *t*(*c*_*max*_). Finally, the cost function *f*_*c*_ (see Equation 32) is minimized to obtain a time stamp *t*_*eucl*_ for each IDC. The transit time can be calculated as the difference of both time stamps *t*_*eucl*_ of one data set.

(32) $$\begin{array}{l} f_{c}\left(t_{eucl}\right)=\sqrt{{\left(\frac{f_{model}\left(t_{eucl}\right)}{c_{max}}\right)}^{2}+{\left(\frac{t_{eucl}-t\left(c_{max}\right)}{t_{end}-t\left(c_{max}\right)}\right)}^{2}} \end{array}$$

#### 2.2.6. Control Group

A control group is added to enable the assessment of the performance of the proposed methods. Using the peak of the raw data relies on a single sample point in each IDC and is very noise sensitive. The cross-correlation of the IDCs relies on a larger set of sample points is less noise sensitive and therefore used for this performance assessment. The cross-correlation is applied to the raw input data *c*(*t*), its first derivative Δ*c*(*t*) (discrete sample to sample difference), the linear interpolated input data *c*_*interpolate*_(*t*), and to the interpolated first derivative Δ*c*_*interpolate*_(*t*). A linear interpolation with a factor of 100 is applied to enhance the accuracy.

In total, 22 combinations of mathematical models and features as well as 4 control groups are used to assess the transit time error on all 12,096 *in silico* generated data sets.

#### 2.2.7. Performance Parameter

The performance parameter used to compare different methods ascertaining the transit time was defined as the absolute difference between the measured transit time Δ*t*_*calc*_ of two IDCs of one data set and the ground truth transit time Δ*t*. It will be represented as ε_*frames*_ in frames to emphasize whether a sub-frame rate accuracy is accomplished or not. The calculation of the error in frames ε_*frame*_ is shown in Equation (34).

(33) $$\begin{array}{l} \varepsilon =|\Delta t_{calc}-\Delta t_{Ground\;truth}| \end{array}$$

(34) $$\begin{array}{l} \varepsilon_{frame}=|\Delta t_{calc}-\Delta t_{Ground\;truth}|\cdot f_{sampling} \end{array}$$

The mean value μ and standard deviation σ of ε_*frame*_ will be used for the evaluation. The μ and σ are calculated using 12,096 data sets

- 3 morphologically different IDCs;
- 7 different SNR levels;
- 6 cycles for each SNR level;
- 4 different transit times Δ*t*;
- 2 different sampling rates *f*_*sampling*_;
- 12 different combinations of sub-sampling.

for each combination of methods and pre-processing (546 in total)

- 26 combinations of different mathematical models, features, and control group;
- 3 methods of cutting the data;
- 7 levels of cutting the data.

The absolute error at a certain SNR ε(*SNR*) should decrease with an increasing sampling rate. The ratio of decrease in ε(*SNR*) is the same as the ratio of the increase in sampling rate. An aspect that is not considered in this evaluation is the increasing effect of noise on the measured signal due to a shortened integration time (exposure time) at higher sampling rates at the detector. This effect can be simulated by correcting the SNR by multiplying it with the square root of the ratio of the two sampling rates. This approach assumes uncorrelated noise and a linear decrease in integration time with increasing sampling rate.

## 3. Results

The following two sections show the results for the generation of the data sets containing two corresponding IDCs with a ground truth of the transit time Δ*t* (section 3.1) and their analysis (section 3.2) separately.

### 3.1. Results Generation

**Figure 6:**
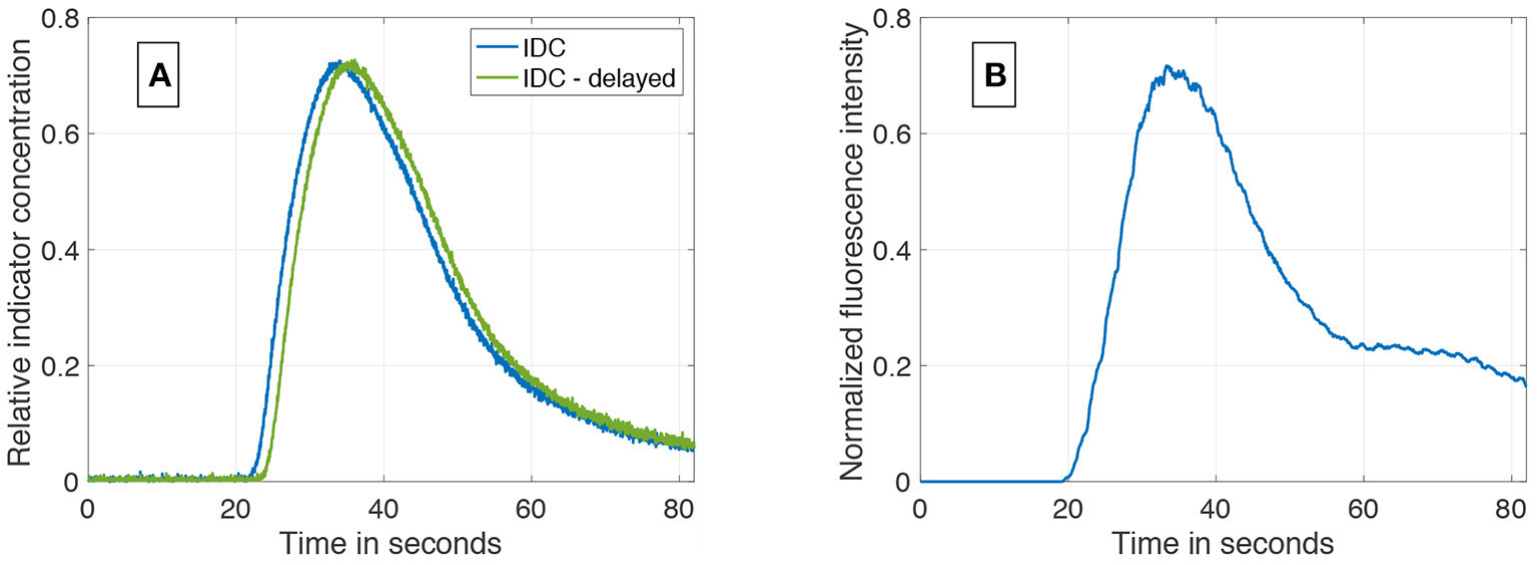
**(A)** An example of a synthetically computed data set. It shows two indicator dilution curves with an delay of 1.5 *s* and a SNR of 37 *dB*. **(B)** An *in vivo* indicator dilution curve obtained from a cerebrovascular EC-IC bypass surgery by indocyanine green fluorescence angiography. The signal is obtained by averaging an area of 10 × 10 pixel which corresponds to one-third of the vessel's diameter.

**Figure 7:**
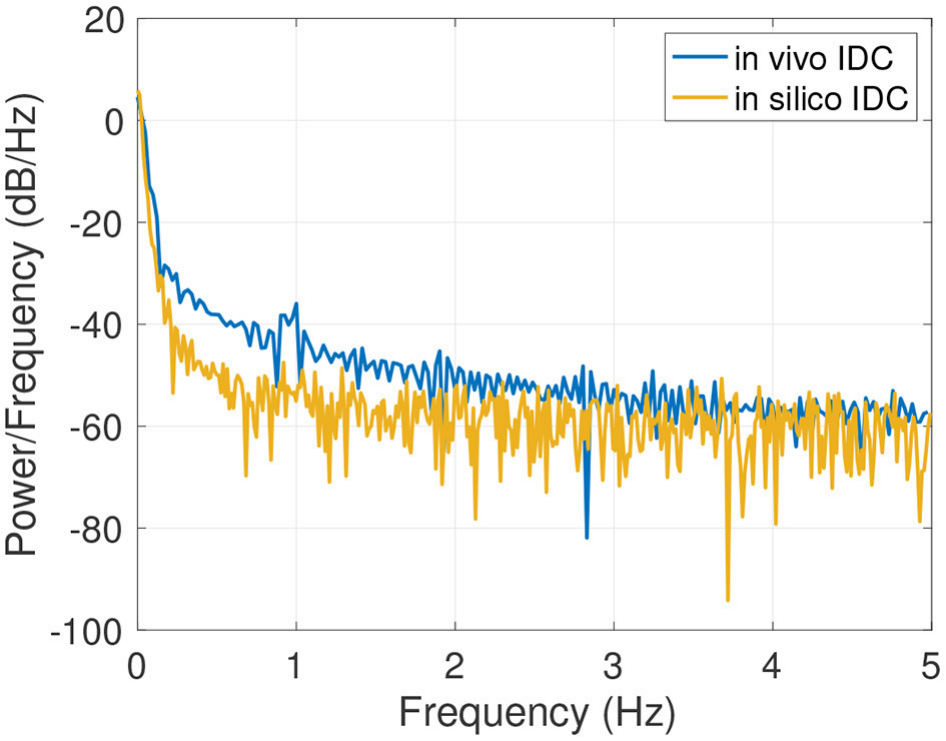
Power spectral density of the *in vivo* and *in silico* data in the spectral rang of up to 5 *Hz*.

An example of the results of the data generation is shown in Figure 6A. The generated IDC has a similar morphology as the clinically obtained *in vivo* IDC in Figure 6B. The Pearson's correlation coefficient is 0.93. Since the *in silico* model does not include a circulatory flow, no recirculation appears in the generated data set compared to the *in vivo* data set. Figure 7 shows the power spectral density for the *in vivo* and *in silico* data set. The frequency is cut off after 5 *Hz* because it does not contain any significant changes. The *in vivo* data are obtained during an EC-IC bypass installation by a surgical fluorescence microscope in high definition resolution, with a sampling frequency of 60 *fps*.

### 3.2. Results Analysis

The mean computation time from the input of two IDCs to the output of the transit time error was ~0.298 *s*. This includes the initial estimation of the mathematical functions' parameters *p*_*init*_ for both IDCs, the optimization of the parameters and finally the determination of the temporal delay *t*_*calc*_ as well as the comparison to the ground truth transit time Δ*t* to evaluate the temporal accuracy ε and ε_*frame*_.

**Figure 8:**
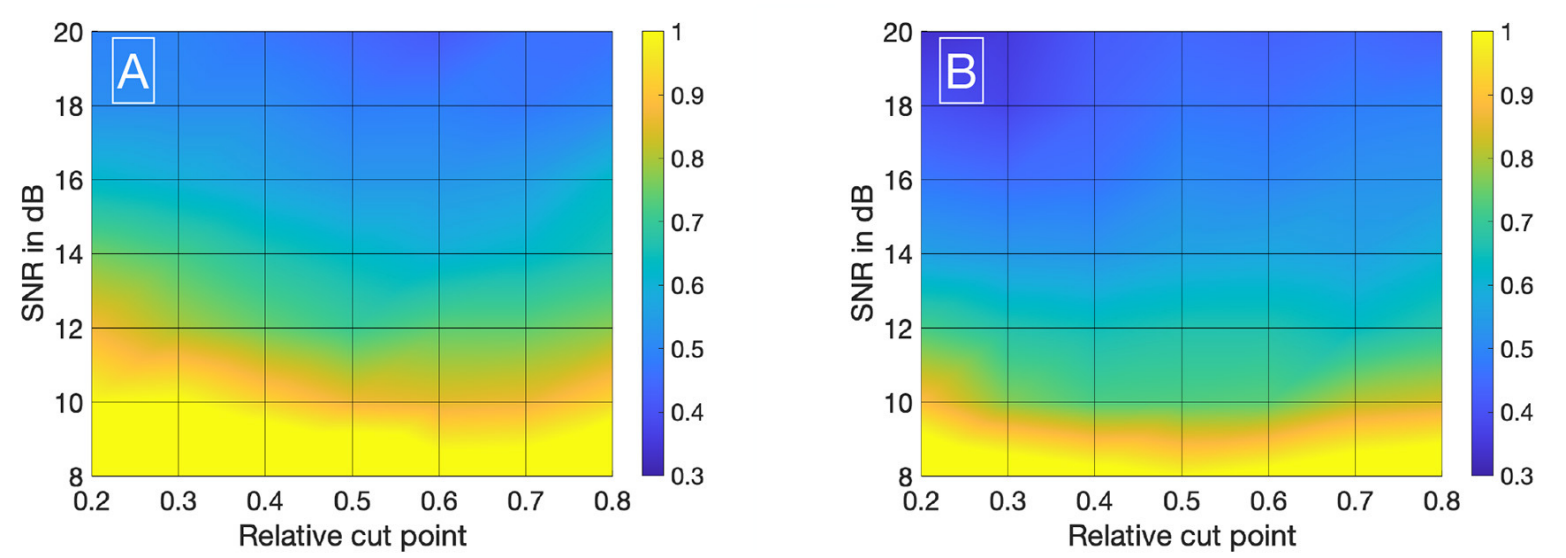
**(A)** Mean ε_*frames*_ using the raw data sets (*f*_*sampling*_ = 25 *Hz*) with no mathematical fits. To obtain the transit time, the cross-correlation is computed after applying the cut method 3. **(B)** Mean ε_*frames*_ using the linearly interpolated data sets (*f*_*sampling*_ = 25 · 100 *Hz*) with no mathematical fits. To obtain the transit time, the cross-correlation is computed after applying the cut method 2.

**Figure 9:**
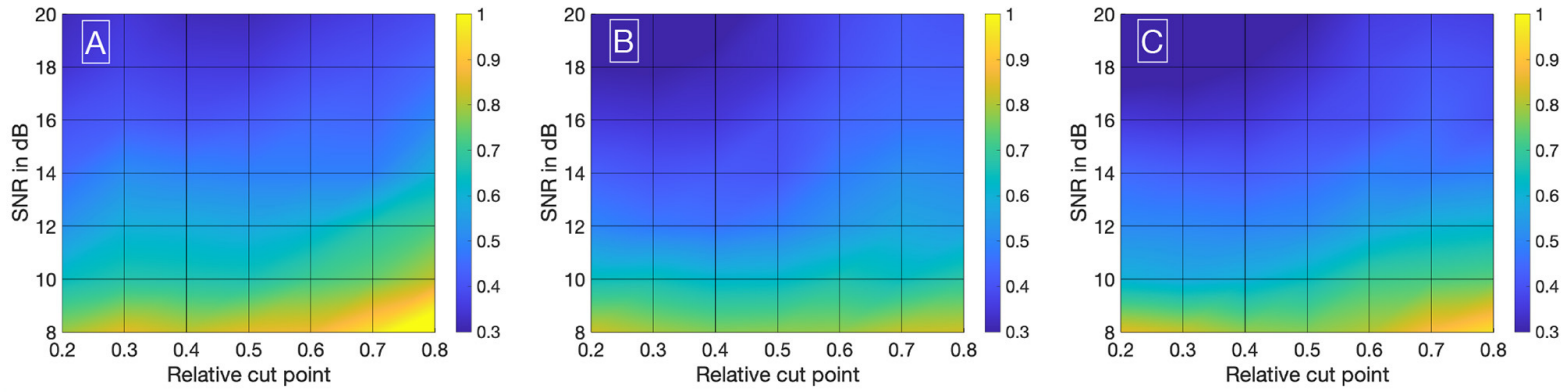
**(A)** Mean ε_*frames*_ using the gamma variate model on the data sets (*f*_*sampling*_ = 25 *Hz*). Before fitting the model, cut method 3 is applied to the data set. To obtain the transit time, the cross-correlation is computed. **(B)** Mean ε_*frames*_ using the gamma variate model on the data sets (*f*_*sampling*_ = 25 *Hz*). Before fitting the model, cut method 1 is applied to the data set. To obtain the transit time, the cross-correlation of the first derivative is computed. **(C)** Mean ε_*frames*_ using the LDRW model on the data sets (*f*_*sampling*_ = 25 *Hz*). Before fitting the model, cut method 1 is applied to the data set. To obtain the transit time, the cross-correlation of the first derivative is computed.

**Figure 10:**
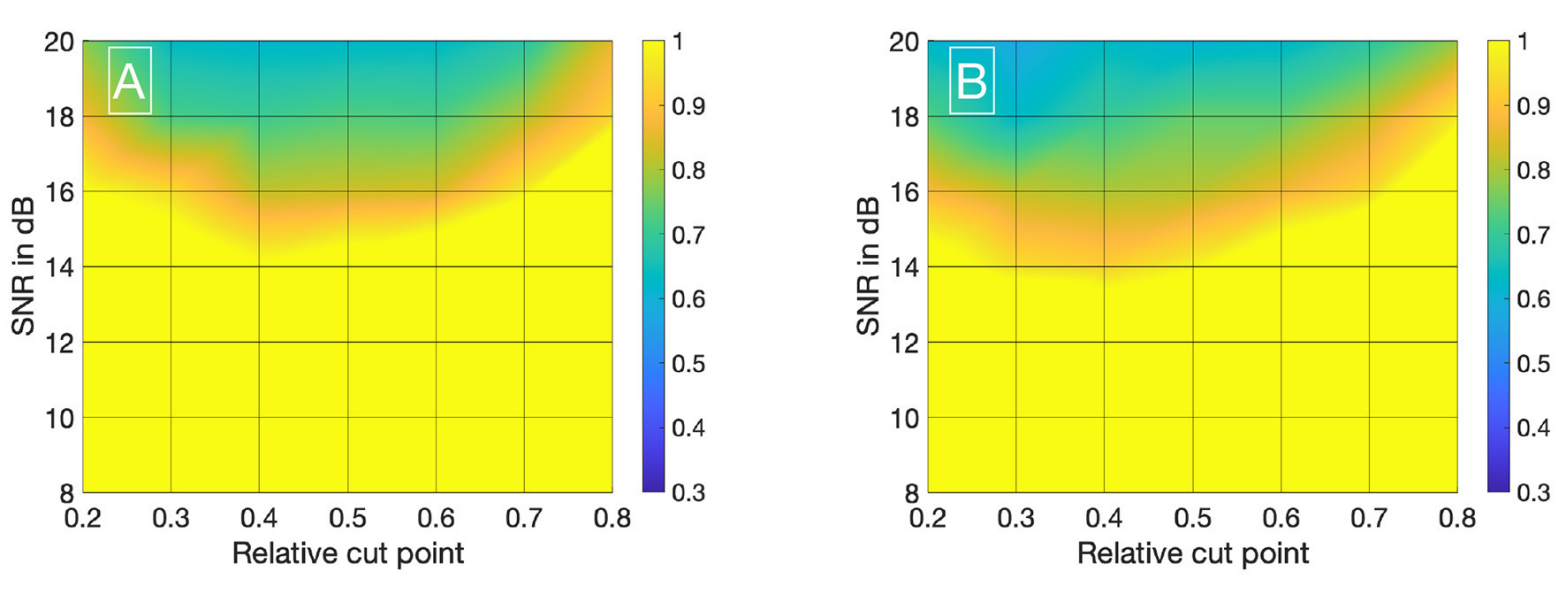
**(A)** Mean ε_*frames*_ using the raw data sets (*f*_*sampling*_ = 60 *Hz*) with no mathematical fits. To obtain the transit time, the cross-correlation is computed after applying the cut method 2. **(B)** Mean ε_*frames*_ using the linearly interpolated data sets (*f*_*sampling*_ = 60 · 100 *Hz*) with no mathematical fits. To obtain the transit time, the cross-correlation is computed after applying the cut method 2.

**Figure 11:**
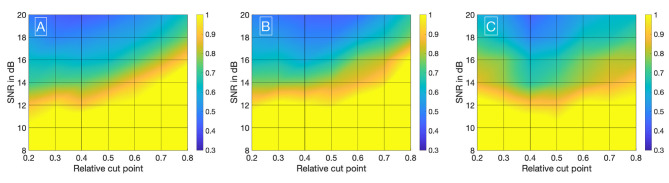
**(A)** Mean ε_*frames*_ using the gamma variate model on the data sets (*f*_*sampling*_ = 60 *Hz*). Before fitting the model, cut method 3 is applied to the data set. To obtain the transit time, the cross-correlation is computed. **(B)** Mean ε_*frames*_ using the LDRW model on the data sets (*f*_*sampling*_ = 60 *Hz*). Before fitting the model, cut method 3 is applied to the data set. To obtain the transit time, the cross-correlation is computed. **(C)** Mean ε_*frames*_ using the LDRW model on the data sets (*f*_*sampling*_ = 60 *Hz*). Before fitting the model, cut method 1 is applied to the data set. To obtain the transit time the cross-correlation of the first derivative is computed.

Visualizing all 8,036 combinations of methods ascertaining the shift of two IDCs is not appropriate. To facilitate the visualization of the results, a 7 × 7 matrix is used to show the mean ε_*frame*_. The values for the mean ε_*frame*_ are determined for the values labeled at the axis, the space in between is interpolated. Each matrix is specific for a combination of a mathematical function, feature, cutting method, and sampling frequency *f*_*sampling*_ and shows the mean ε_*frame*_ in dependency of the preformed cutting levels as a pre-processing step and the SNR. The color code in Figures 8–11 represents ε_*frame*_ and errors larger than 1 are presented in yellow. The performance of the cross-correlation on the raw data set with *f*_*sampling*_ = 25 *fps* is shown in Figure 8A as benchmark for the proposed methods. The linear interpolation of the raw data already shows a decrease of the error (Figures 8A,B). The best performance from the control group at *f*_*sampling*_ = 25 *fps* was the cross-correlation of the linearly interpolated data set with the cutting method 2 (cutting at half the value left of the peak compared to the variable cutting to the right of the peak) and is shown in Figure 8B. The results of the three best combinations of mathematical functions and features ascertaining the transit time with the data set at *f*_*sampling*_ = 25 *fps* are shown in Figures 9A–C. They all perform equally well and show a strong decrease in error compared to the usage of the raw data (Figure 8B). The results at *f*_*sampling*_ = 60 *fps* are shown in Figures 10, 11 and show a similar performance compared to the lower sampling frequency. The methods that perform best at 25 *fps* also tend to perform best at 60 *fps*.

In Supplementary Material (sections 2, 3), you can find the corresponding values for μ and σ in tabular form used to compute Figures 8A–11C.

## 4. Discussion and Conclusion

Obtaining the ground truth transit time of two corresponding indicator dilution curves is a challenge. We have proposed and implemented a method to simulate adjustable IDCs with a known ground truth of the transit time in a statistical relevant quantity. Thereby, a high degree of freedom in the variation of the signal's morphology, sampling rate, sample positioning, transit time, and noise level is given. This allows a versatile use of the *in silico* model to mimic different signals obtained from different organs. As an alternative to COMSOL Multiphysics, other numerical simulation tools can be used as well to simulate the presented IDCs. Without depending on advanced simulation software, analytic methods such as the Taylor–Aris approach exist and need be adapted to the setup by introducing additional perturbation terms as presented by Alizadeh et al. (1980) and Aris (1956).

The presented dilution curves were simulated by an *in silico* model that took the properties of a fluid flow and occurring chemical interactions such as diffusion of the used indicator into account, so the model should be transferable to mimic different non-diffusible indicator types. Note, diffusion occurs in non-diffusible indicators within the solvent media, but not with the surrounding tissue (e.g., a thermal indicator diffuses into the surrounding tissue and is therefore a diffusible indicator, Indocyanine Green remains intravascularly, has nearly no diffusion into surrounding tissue and is therefore a non-diffusible indicator). The chosen diffusion coefficient for ICG *D*_*ICG*_ (see Supplementary Material, section 1.1) is set equal to the diffusion coefficient of the protein. Since ICG binds to the proteins and is at the initial state of the study in equilibrium, we assume it will have the same diffusion coefficient. The proposed *in silico* model uses a laminar flow of a homogeneous liquid in a rigid vessel. Blood is in contrast a cell suspension and vessels are elastic. Assuming blood as a homogeneous fluid influences the dynamics of most fluorophores since most are bound either to the cells or to the plasma. This aspect is not included in the present study. Generally, a transfer to different recording modalities should be handled with care. Assuming a continuous volume flow is motivated by a significantly decreasing Gosling pulsatility index from measurements taken proximal and distal on certain cerebral vessels (Gosling and King, 1974; Zarrinkoob et al., 2016). This significant decrease does not imply the disappearance of the pulsatility and therefore limits the results of this study to distal cerebral arteries. The geometry of the setup represents the *in vitro* flow phantom build in our facility (details to this phantom are given in the Supplementary Material). The generated IDCs match well the measured IDC in the phantom (Pearson's correlation coefficient of 0.98–0.99, see Supplementary Material, section 3.7). This proves its validity in predicting the IDC for this configuration and also validates the second assumption given in section 2. An extensive comparison with *in vivo* data is not possible due to the lack of data. Nonetheless, the presented case shows a high accordance with the simulated data (Pearson's correlation coefficient of 0.93). Both, *in vivo* and *in vitro* comparisons prove the similarity of the IDCs (see Supplementary Material, sections 3.1, 3.7). The *in silico* setup represents the vessel with an open end and no recurrence of the bolus, thus limiting the reliability of data points on the curve's descending side where recirculation does not appear (in Figure 6A, no bump is present after the peak as in Figure 6B). Nevertheless, in most methods ascertaining the transit time Δ*t*, the dilution curve is cut off after its concentration has decreased to 30–50% of the respective peak, which excludes most influences by the recirculation (Millard, 1997; Mischi et al., 2003; Reuter et al., 2010). Furthermore, in the measurement of blood volume flow in cerebrovascular bypass surgery, the distance of both measurement location is very short (< 2 *cm*). Thus, the influence by recirculation will be nearly identical in both signals, equally influencing the two mathematical fits and thereby not influencing the transit time measurement. Modeling the injection as a rectangular input function assumes an ideal abrupt injection. In clinical practice, the anesthetist will inject the indicator and an inter- and intra-individual variability will occur. The injection will deviate from the modeled input function. Still, the indicator will be homogenized in the hearts chamber and afterward ejected into the aorta as a rectangular input function with each heartbeat. Choosing the rectangular input function facilitates and standardizes the study design to ensure a comparability of the results. White Gaussian noise (WGN) is applied to the data set's two corresponding IDCs to represent Johnson–Nyquist noise. However, WGN only approximates Johnson–Nyquist noise and as well does not cover all sources of noise and/or artifacts observed in *in vivo* measurements. Adding other noise sources such as shot noise and artifacts such as the spatial exclusion of the dye by blood cells requires a more profound investigation on *in vivo* data. Nonetheless, the robustness of the proposed algorithms can be evaluated using WGN. The example in Figure 6B shows distinctive noise/artifacts in the area of the peak concentration, which seems to be not reproduced by WGN. The presented *in vivo* signal is an average of an area of 10 × 10 pixel uncorrelated noise, such as WGN, is reduced. Correlated noise and artifacts will remain present in the signal. The ripples on the signal shown in Figure 6B are at a frequency of approximately 1 *Hz* and might be the patient's pulse, which is a correlated noise source. This cannot be verified since no vital monitoring data are available. Nevertheless, the simulated curves and their power spectral density (Figure 7) show a high accordance with the clinically obtained *in vivo* data from intraoperative fluorescence angiography measurements. Performing an objective comparison is possible but is not expedient since the morphology of the available *in vivo* data is limited. The transfer function from the dye's concentration to the optically measured fluorescence is not known and has an influence on the optically measured signal since it affects the signals morphology. In this paper, we focus on the measurement error and assume the effect of the transfer function as negligible. The presence of a transfer function would induce a systematic error to the measurement, which can be separated from the investigation performed in this paper. Therefore, we suppose these data sets are suitable for the evaluation of methods ascertaining the transit time of optically measured indicator dilutions curves.

**Figure 12:**
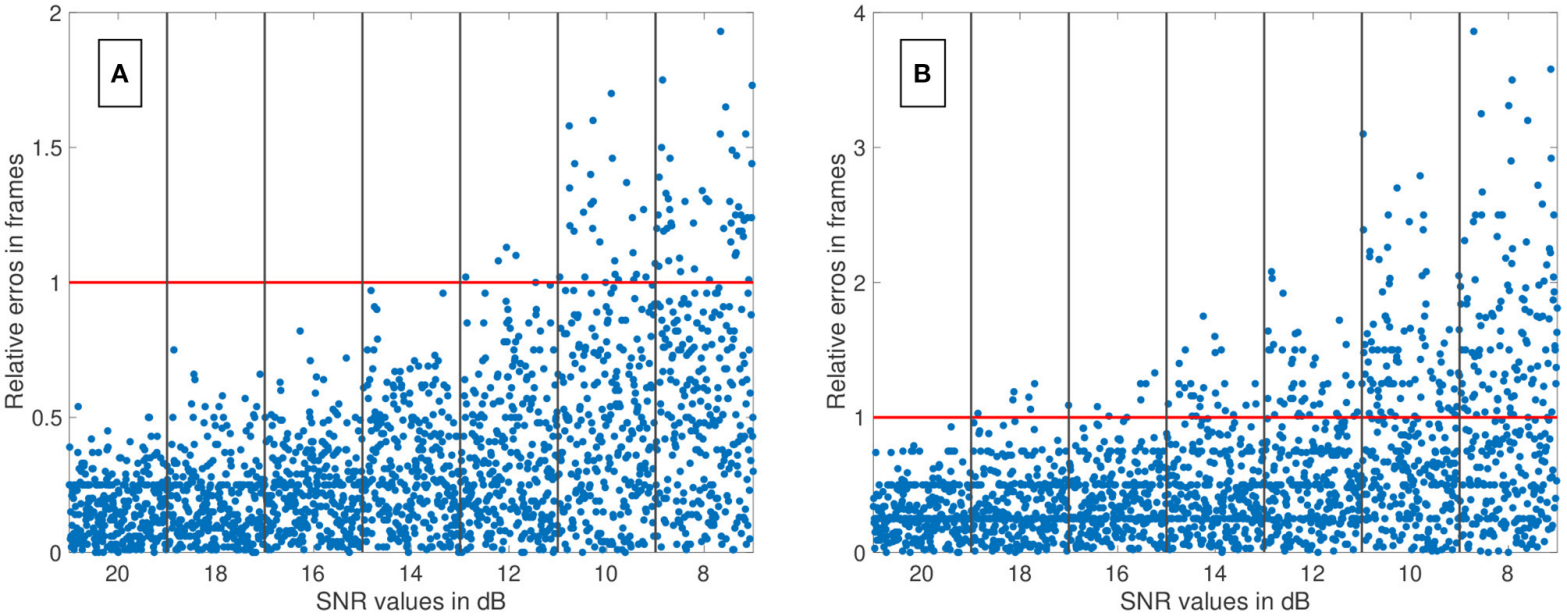
**(A)** The relative error of all data sets (*f*_*sampling*_ = 25 *fps*) is shown in relation to the signal-to-noise ratio (SNR). The red line indicates the relative error of one frame. The blue dots show the single results obtained by the fitting the Local Density Random Walk (LDRW) model to the data cut at 40% by the cut method 1 and using its first derivative as input for the cross-correlation. The distribution of the dots within a column is irrelevant. All data sets had a relative error of less than one frame for SNR larger than 12 *dB*. **(B)** The relative error of all data sets (*f*_*sampling*_ = 60 *fps*) is shown in relation to the SNR. The red line indicates the relative error of one frame. The blue dots show the single results obtained by the fitting the LDRW model to the data cut at 40% by the cut method 1 and using its first derivative as input for the cross-correlation. The distribution of the dots within a column is irrelevant. All data sets had a relative error of less than one frame for SNR larger than 18 *dB*.

We have evaluated the performance of different methods ascertaining the transit time with varying complexity. Thereby, the transit time was calculated according to the systemic mean transit time theorem for single input and single output systems as defined by Perl et al. (1975). This theorem can be used since the shape of the curve does not change significantly due to the short distance between the measurement sites. The first hypothesis that using a mathematical model decreases the error in ascertaining the transit time can be verified with some limitations. Obviously, not all combinations of mathematical models and features are suitable for the enhanced measurement of the transit time of an indicator bolus. Especially the mono-exponential function showed a bad performance, probably due to a complex feature-based metric measuring the delay. The gamma variate and the LDRW model performed equally well. Both are suitable to verify the hypothesis especially using the cross-correlation that takes all data points into account and not solely the peak to peak distance. Verification of the second hypothesis that a sub-frame rate accuracy can be accomplished by using a suitable configuration of mathematical function and pre-processing in combination with a feature is also limited. For a sampling frequency of 25 *Hz* the configuration: gamma variate, cross-correlation of the first derivative using an up-sampling factor of 100 and cut method 1 performed best. It showed a mean value for ε_*frame*_ of <0.85 for all SNR (8–20 dB) and cut levels (see Figure 9B). The Figures 12A,B also underline its robustness. Please note that the line like artifacts are a visualization issue of this plot. In case of a *f*_*sampling*_ = 25 *fps*, a sub-frame rate accuracy was accomplished in all cases as soon as the noise level was above a *SNR* ≥ 14 *dB*. Accomplishing a sub-frame rate accuracy with a higher sampling frequency is harder since the absolute time tolerance decreases. The appearance of a lower performance in case of a higher sampling rate is misleading. The reduction of the absolute time error ε(*SNR*) has a mean value of ~26% lower at 60 *fps* than in 25 *fps* (see Table 1). Assuming no noise the accuracy should increase by the ratio of the sampling rates, here $1-\frac{25}{60}=60.3\%$. This shows that noise has a clear influence on the sampling rate dependent decrease of ε. Nevertheless, the influence of a decrease of SNR with increasing sampling rate (so decreasing integration time) is not included in this evaluation and therefore the reduction of the error by 26% is an optimistic calculation. The evaluation of more and different mathematical models, such as the log-normal and lagged-normal model, is possible and could reveal more suitable models (Strouthos et al., 2010).

**Table 1:** Mean absolute error ε and its standard deviation for two sampling frequencies *f*_*sample*_ in dependency of the signal-to-noise ratio (SNR).

**SNR**	**25 *fps***	**60 *fps***
20 dB	10.0 ± 7.1 ms	7.2 ± 5.3 ms
18 dB	12.1 ± 7.9 ms	8.3 ± 6.4 ms
16 dB	13.9 ± 9.5 ms	10.7 ± 8.8 ms
14 dB	16.8 ± 12.1 ms	11.4 ± 9.4 ms
12 dB	19.9 ± 15.1 ms	15.3 ± 12.0 ms
10 dB	23.9 ± 18.5 ms	18.5 ± 13.7 ms
8 dB	31.3 ± 22.9 ms	23.5 ± 16.9 ms

*The ground truth transit time was evenly distributed ranging from* 40 *ms to* 160 *ms for f_sample_* = 25 *fps and from* 33 *to* 133 *ms for f_sample_* = 60 *fps*.

Maintaining and restoring a sufficient blood flow is a crucial aspect in preventing post-surgical complications such as cognitive impairment (Lawton and Lang, 2019). Optic-based contact free blood volume flow measurement have distinctive advantages and disadvantages in their handling and performance compared to other techniques. Providing surgeons multiple different blood flow measurement techniques would most probably have a positive impact on the mortality and recurrence rate in revascularization surgery since the quality of the procedure can be checked intraoperatively and worsening of the flow/perfusion can be prevented (Chen et al., 2018). The transit time of a bolus is one of the measured parameters needed for the calculation of the volume flow (Equation 1). Knowing the limits of the measurement quantitatively is key to ensure a safe handling and proper quality of the results. Now, the findings of this paper allow a quantification and reduction of the absolute statistical error in transit time measurement in dependency of the noise level and sequentially its propagation on the volume flow calculation. Assuming a transit time of four frames and a determined error of 0.25 frames for a sampling rate of 25 *fps* and a SNR of 8 *dB* would result in an error in transit time measurement of ~6%, the error in geodesic measurements (according to Equation 1) would be added to this value (Naber et al., 2020a). This error seems to be acceptable in comparison with the accuracy of the state-of-the-art clinical flow probes (Transonic, 2019). The systemic error in optical transit time measurement is not included in this paper but it is an important aspect, which is in focus of our research (Naber et al., 2020b). Its influence in optical blood volume flow measurement is not yet revealed. Nevertheless, this is a large step toward an accurate and intraoperative optical assessment of blood volume flow with the possibility to provide an uncertainty level to the measurement to each case individually. Furthermore, the transit time can be used solely for the relative assessment of the flow before and after an intervention in the cases where no vessel is attached for revascularization (such as in aneurysm clipping).

## 5. Outlook

The proposed hypotheses are verified with some limitations regarding the methods used and present noise level in the signal. The investigated mathematical models and features represent a fraction of the possible methods. Therefore, more mathematical models and features can be implemented and tested on the generated data set. The investigated noise levels are chosen reasonably according to our experience but could be expanded to a larger range and finer gradation. A reduction of the noise has a large impact on the accuracy ascertaining the transit time, so different noise reduction techniques should be considered and evaluated. Nevertheless, care should be taken since the cross-correlation is depending on the data sets' morphology and obviously the morphology is affected by temporal filtering and thereby might affect clinical decision making (Lenis et al., 2017). The absolute error ε in transit time measurement decreased with increasing sampling frequency. So, increasing the frame rate increases the accuracy, but at some point, the benefit will be eliminated by an increased noise level (noise is mostly coupled reciprocally with the integration time). Therefore, we hypothesize that there is a recording modality and purpose specific optimum of the sampling rate with regard to the error in transit time measurement. Additionally, it was assumed that the transfer function from the dyes concentration to the optical measurement is negligible. This transfer function should be obtained and added to the error propagation assessment as a source for a systemic error. Finally, the investigation of methods on *in silico* data is linked to assumptions, which reduce the complexity of *in vivo* data to a manageable level. Therefore, *in vivo* data should be obtained to check the robustness of these methods in real-life scenarios.

## Data Availability Statement

The raw data supporting the conclusions of this article will be made available by the authors, without undue reservation.

## Ethics Statement

The research related to human use complies with all the relevant national regulations, institutional policies and was performed in accordance with the tenets of the Helsinki Declaration, and has been approved by the authors' collaborators institutional review board or equivalent committee.

## Author Contributions

AN and WN conceptualized the research. AN, MR, and WN designed the research. AN and MR organized the software and database. MR performed the statistical analysis. AN wrote the first draft of the manuscript. All authors contributed to manuscript revision, read, and approved the submitted version.

## Conflict of Interest

The authors declare that the research was conducted in the absence of any commercial or financial relationships that could be construed as a potential conflict of interest.
