# Supplementary material for: Transit Time Measurement in Indicator Dilution Curves: Overcoming the Missing Ground Truth and Quantifying the Error
Source: Front Physiol. 2021 May 28;12:588120. doi: 10.3389/fphys.2021.588120 (PMC8194354; doi:10.3389/fphys.2021.588120)
Supplement: Supplementary file 1 [file Data_Sheet_1.ZIP › Supp_Mat/KIT-Poster_en_2015.pdf]

Karlsruhe Institute of Technology

Institute of Biomedical Engineering
Kaiserstraße 12 · D-76131 Karlsruhe · www.ibt.kit.edu

Design of a Flow Phantom for the Evaluation of Quantitative ICG Fluorescence Angiography

Ady Naber, Leila Meyer-Hilberg, Werner Nahm

Motivation

- Intraoperative quality check in neurovascular surgical interventions (e.g. bypass) is done subjectively by the surgeon with the help of Indocyanine Green (ICG) fluorescence angiography
- Research focussed on quantitative assessment of flow (ml/s)
- Standardized and validated flow phantom is needed to assess optical methods performance and a digital twin to obtain the ground truth concentration values

Therefore we design a flow phantom and a digital twin suitable for the evaluation of quantitative ICG Fluorescence Angiography [1]

Methods

- Requirements on the Phantom
  - Variable diameter of tubes/pipes mimicking cerebral vessel (~1-5mm)
  - Variable flow within the clinical relevant ranges for cerebral vessels
  - Handy and precise injection system of the ICG
  - Non toxic and low cost blood analog with similar viscosity and binding properties as human blood
  - Gold standard flow and pressure measurement
  - Ensure standardized conditions at the investigation site, no mixing of ICG Solution with others than the blood analog
- COMSOL Multiphysics simulation
  - Use of CFD & CRE (Computational Fluid Dynamics, Chemical Reaction Engineering) with 3 physic interfaces: laminar flow, transport of concentrated and diluted species
  - Digital twin of phantom to obtain spatially resolved dye concentrations
  - Simulation of the mixing and diffusion of ICG within the phantom to ensure the last requirement
    - For the diameters 1, 2, 3, 4, 5 mm
    - Lower and upper physiological flow values realizable with the pump
  - Acceptance criteria
    - ICG solution should not mix with water, since the fluorescence properties are depending on the solvent
    - Therefore ICG bolus should stay within the blood analog bolus

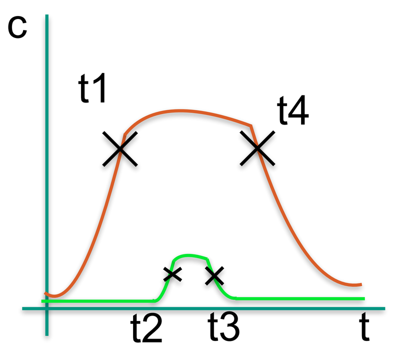

$$\tau_1 = t_2 - t_1 \qquad \tau_2 = t_4 - t_3 \qquad \overset{!}{\tau_1 > 0} \qquad \overset{!}{\tau_2 > 0}$$

Figure 1: Definition of the threshold as 0.9 of the initial concentration or maximum concentration

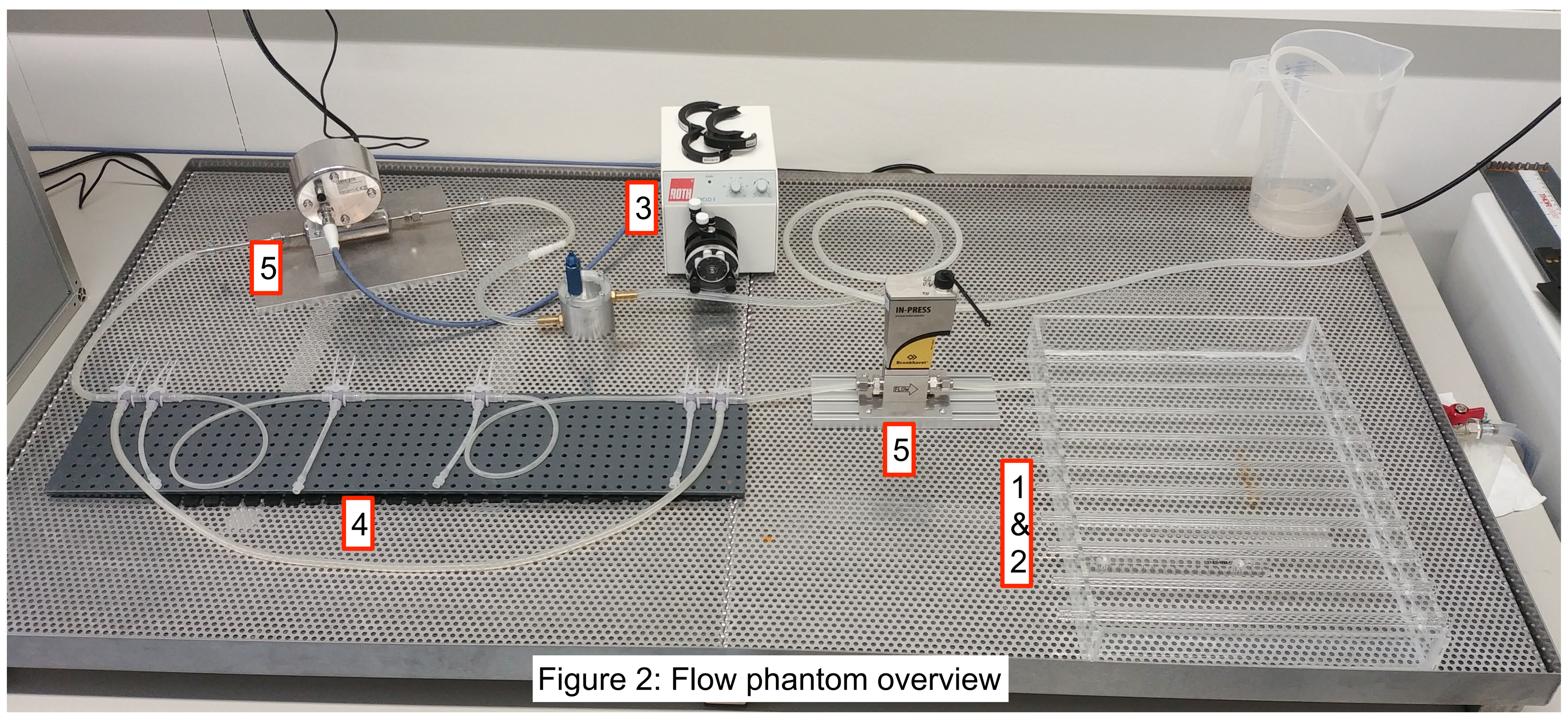

Figure 2: Flow phantom overview

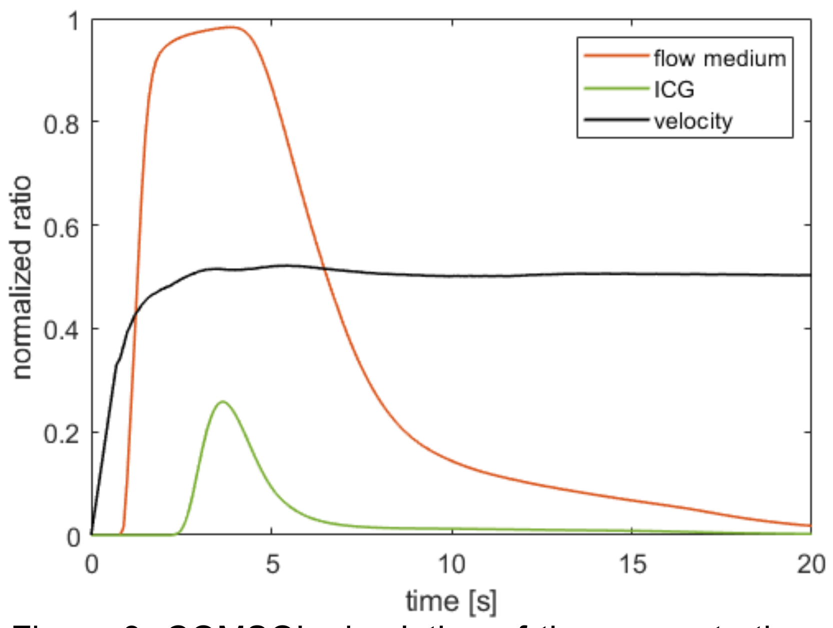

Figure 3: COMSOL simulation of the concentration of the flow medium, ICG (relativ to the initial c) and the mean flow velocity, here the acceptance criteria are fulfilled

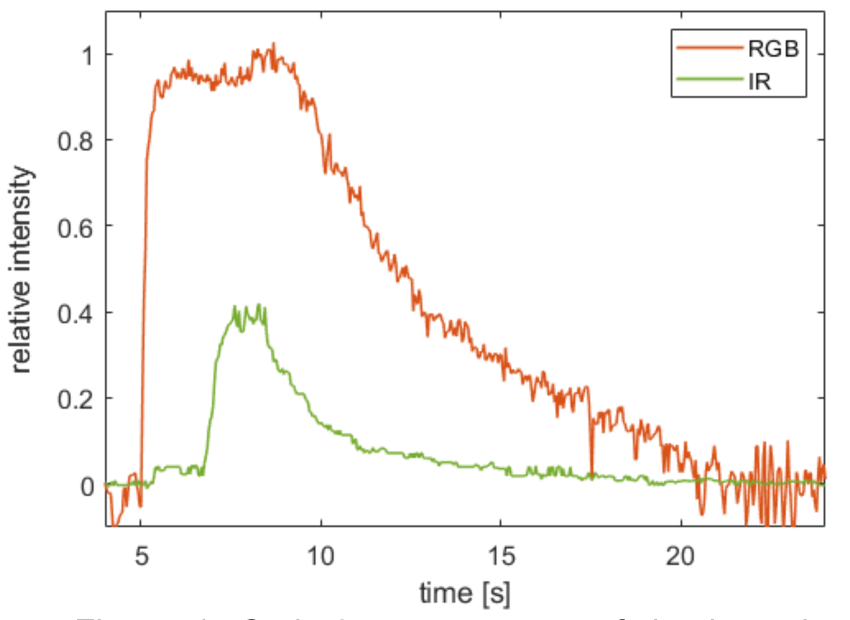

Figure 4: Optical measurement of the intensity, RGB represents the flow medium, in IR the ICG is represented. Subjectively it complies with the COMSOL simulation

Results

- Flow Phantom (Figure 2)
  1. Silicon tubes: 1mm to 10mm inner diameter
  2. Glas tubes: 0.8mm to 10mm inner diameter in a index matching bath to reduce reflexes
  3. Pump providing continuous or pulsatile flow from 10 ml/min to 200 ml/min, 40 to 120 bpm, 0 to 400 mmHg
  4. Bypass system to inject rectangular dye signals into the system by 3-way selector valves
  5. Industrial flowmeter and pressure sensor (Bronkhorst ES113 and EL-PRESS)

  X. Blood analog with a blood matching viscosity mixed from demineralized water, Glycerol, protein powder and Xanthan
- COMSOL Multiphysics simulation
  - Spatially resolved and quantitative dye concentration distributions in the tubes are obtained
  - The lower and upper boundary of the investigation sites where the dye-enriched analog does not mix with the water is:

Inner diameter [mm]	Volume flow [ml/min]	Lower boundary [m]	Upper boundary [m]
1	5	0	1
	7	0	1
2	19	0	1
	94	0	1
3	42	0	1
	297	0	1
4	151	0	0.6
	377	0	0.6
5	236	0	0.2
	471	-	-

Table 1: Lower & upper boundary in dependance of the inner diameter and minimum and maximum flow

- COMSOL simulation and optical measurement agree with each other (Figure 3 &4)

Discussion & Conclusion

- Flow phantom can be used to assess the performance of algorithms used for quantitative fluorescence angiography
- Ground truth data of the flow and pressure can be recorded and analyzed automatically
- Various diameters and flow values (continuous and pulsatile) can be controlled and reflexes at the investigation site are attenuated by matching the refractive index of the surrounding media
- Investigational site and ICG-bypass injector design are matched using COMSOL to ensure no mixing or diffusion of ICG with others than blood analog
- Quantitative ground truth dye concentrations are calculated in COMSOL
- COMSOL simulation in combination with the phantom can be used for setting up ICG concentration and image intensity relations
- The phantoms and simulations design is modular and can be easily extended

Outlook

- Relation of ICG concentration and image intensity can be investigated and set into relation
- Flow phantom will be used to evaluate algorithms measuring the flow of the medium
- Comparisons of continuous and pulsatile flow measurements with different flows and pressure values will be done
- Different vessel models (bifurcation, stenosis etc.) can be easily connected to the phantom (Plug & Play)
- Glycerol to water ratio can be varied to change the viscosity of the blood analog

References

[1] - C. Weichelt et al., "Development of a Flow Phantom to Verify the Evaluation of Cerebral Blood Flow by ICG-Fluorescence Video Analysis" IFMBE Proceedings, vol. 25/IV, pp. 1448-1451, 2009.

Funding

This research is funded by the Carl Zeiss Meditec AG

Ady Naber
+49-721-608-42751
ady.naber@kit.edu
