# Supplementary material for: Transit Time Measurement in Indicator Dilution Curves: Overcoming the Missing Ground Truth and Quantifying the Error
Source: Front Physiol. 2021 May 28;12:588120. doi: 10.3389/fphys.2021.588120 (PMC8194354; doi:10.3389/fphys.2021.588120)
Supplement: Supplementary file 1 [file Data_Sheet_1.ZIP › Supp_Mat/frontiers_SupplementaryMaterial_revisedV4.pdf]

Supplementary Material

1 SUPPLEMENTARY DATA

1.1 COMSOL Multiphysics

The *in silico* computation of the presented raw indicator dilution is performed in COMSOL Multiphysics V5.4. You can find the .mph file in the folder Supplementary Material. Following is an overview of the parameters defined in the simulation:

- Initial indicator concentration $c_{ICG} = 6.4519 \, \frac{mmol}{m^3} \; (= 0.005 \, \frac{g}{l})$
- Diffusion coefficient D based on (Kinsella and Whitehead, 1989; Tyn and Gusek, 1990):
  - $D_{ICG} = 7.6 \cdot 10^{-11} \, \frac{m^2}{s}$
  - $D_{Water} = 2 \cdot 10^{-9} \, \frac{m^2}{s}$
  - $D_{Glycerol} = 1.37 \cdot 10^{-12} \, \frac{m^2}{s}$
  - $D_{Protein} = 7.6 \cdot 10^{-11} \, \frac{m^2}{s}$
- Density ϱ
  - $\varrho_{Water} = 1009 \, \frac{kg}{m^3}$
  - $\varrho_{Glycerol} = 1263 \, \frac{kg}{m^3}$
  - $\varrho_{Protein} = 1087 \, \frac{kg}{m^3}$
  - $\varrho_{Blood\,Analog} = 1157 \, \frac{kg}{m^3}$
- Molar mass M
  - $M_{ICG} = 0.7496 \, \frac{kg}{mol}$
  - $M_{Glycerol} = 0.092094 \, \frac{kg}{mol}$
  - $M_{Water} = 0.018015 \, \frac{kg}{mol}$
- Reynolds number Re
  - $Re_{Blood\,Analog} = 79.613$
  - $Re_{Water} = 808.29$
- Dynamic viscosity η
  - $\eta_{BloodAnalog}(\dot{\gamma}) = 0.00058 \cdot (1 - 0.69)^{-2.5} + \frac{0.625^3 \cdot 0.69^3}{10 \cdot \dot{\gamma}} \, mPa \cdot s$, modeled by the Casson-model
  - $\eta_{Water} = 1 \, mPa \cdot s$

2 RESULTS AT 25 FPS: FIGURES 8A - 9C IN THE MANUSCRIPT

2.1 Cross-correlation of the raw data sets cut by method 3 - Figure 8A

Figure S1: Figure 8A in the manuscript: mean ε_{frames} using the raw data sets ($f_{sampling} = 25\,Hz$) with no mathematical fits. To obtain the transit time the cross-correlation is computed after applying the cut method 3.

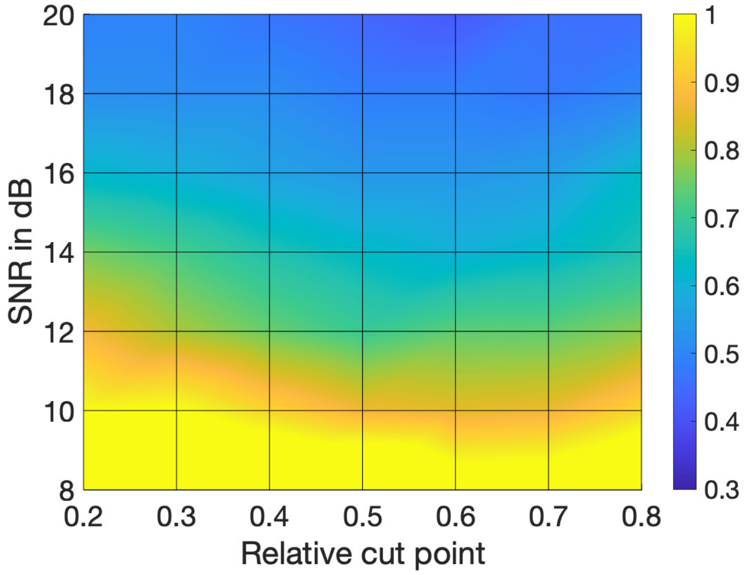

**μ in frames**							
	**Cut level in % of C_{max}**						
**SNR**	**20%**	**30%**	**40%**	**50%**	**60%**	**70%**	**80%**
**20 dB**	0,49	0,49	0,47	0,44	0,42	0,46	0,46
**18 dB**	0,52	0,51	0,51	0,48	0,49	0,47	0,50
**16 dB**	0,62	0,60	0,56	0,53	0,53	0,55	0,63
**14 dB**	0,76	0,72	0,66	0,63	0,60	0,62	0,66
**12 dB**	0,87	0,80	0,75	0,70	0,74	0,74	0,77
**10 dB**	1,00	1,01	0,93	0,87	0,85	0,86	0,94
**8 dB**	1,31	1,23	1,19	1,19	1,07	1,08	1,20
**σ in frames**							
**20 dB**	0,27	0,28	0,26	0,24	0,23	0,25	0,26
**18 dB**	0,34	0,32	0,31	0,30	0,29	0,28	0,32
**16 dB**	0,42	0,41	0,35	0,34	0,36	0,36	0,41
**14 dB**	0,53	0,51	0,44	0,42	0,41	0,43	0,43
**12 dB**	0,62	0,62	0,55	0,53	0,56	0,56	0,56
**10 dB**	0,79	0,75	0,65	0,62	0,64	0,68	0,70
**8 dB**	0,96	0,93	0,89	0,86	0,80	0,81	0,87

Table S1. Top: calculated mean ε_{frame}; bottom: calculated standard deviation σ_{frame} for the results presented in Figure S1 in the supplements and Figure 8A in the manuscript.

2.2 Cross-correlation of the interpolated data sets cut by method 2 - Figure 8B

Figure S2: Figure 8B in the manuscript: mean ε_{frames} using the linearly interpolated data sets ($f_{sampling} = 25 \cdot 100\,Hz$) with no mathematical fits. To obtain the transit time the cross-correlation is computed after applying the cut method 2.

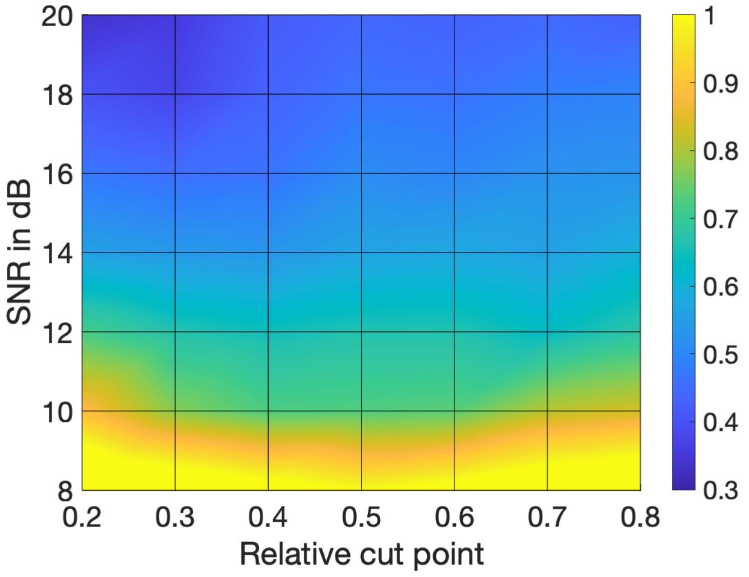

μ in frames							
	Cut level in % of C_{max}						
**SNR**	**20%**	**30%**	**40%**	**50%**	**60%**	**70%**	**80%**
**20 dB**	0,33	0,35	0,42	0,44	0,43	0,45	0,43
**18 dB**	0,40	0,38	0,44	0,47	0,46	0,48	0,49
**16 dB**	0,47	0,45	0,46	0,51	0,50	0,52	0,53
**14 dB**	0,56	0,54	0,53	0,57	0,57	0,56	0,60
**12 dB**	0,72	0,67	0,65	0,67	0,67	0,64	0,68
**10 dB**	0,89	0,77	0,72	0,73	0,74	0,81	0,84
**8 dB**	1,15	1,09	1,04	1,00	1,03	1,09	1,13
σ **in frames**							
**20 dB**	0,23	0,22	0,23	0,24	0,23	0,26	0,25
**18 dB**	0,27	0,24	0,26	0,28	0,27	0,29	0,29
**16 dB**	0,35	0,31	0,30	0,34	0,32	0,33	0,32
**14 dB**	0,44	0,41	0,38	0,36	0,36	0,37	0,41
**12 dB**	0,55	0,50	0,48	0,48	0,46	0,44	0,51
**10 dB**	0,69	0,61	0,54	0,51	0,51	0,58	0,62
**8 dB**	0,86	0,83	0,78	0,72	0,75	0,78	0,83

Table S2. Top: calculated mean ε_{frame}; bottom: calculated standard deviation σ_{frame} for the results presented in Figure S2 in the supplements and Figure 8B in the manuscript.

2.3 Cross-correlation of the fitted Gamma Variate functions cut by method 3 - Figure 9A

Figure S3: Figure 9A in the manuscript: mean ε_{frames} using the Gamma Variate model on the data sets ($f_{sampling} = 25\,Hz$). Before fitting the model, cut method 3 is applied to the data set. To obtain the transit time the cross-correlation is computed.

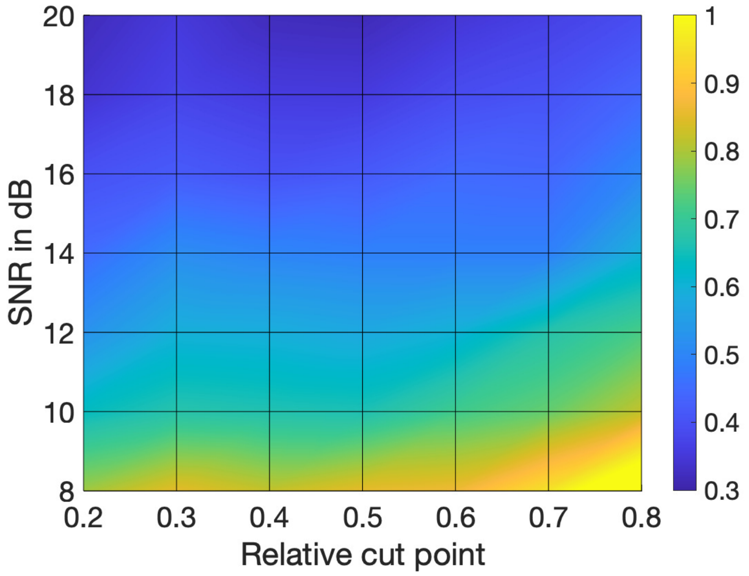

**μ in frames**							
**SNR**	**Cut level in % of C_{max}** 20%	30%	40%	50%	60%	70%	80%
**20 dB**	0,32	0,35	0,32	0,32	0,34	0,37	0,39
**18 dB**	0,34	0,37	0,36	0,36	0,39	0,40	0,44
**16 dB**	0,40	0,42	0,40	0,42	0,45	0,44	0,50
**14 dB**	0,45	0,51	0,50	0,49	0,49	0,49	0,60
**12 dB**	0,53	0,60	0,59	0,58	0,60	0,67	0,72
**10 dB**	0,64	0,67	0,66	0,66	0,71	0,75	0,82
**8 dB**	0,79	0,85	0,82	0,85	0,87	0,96	1,11
**σ in frames**							
**20 dB**	0,19	0,24	0,21	0,21	0,21	0,21	0,24
**18 dB**	0,22	0,24	0,24	0,23	0,25	0,25	0,28
**16 dB**	0,27	0,32	0,27	0,27	0,28	0,30	0,35
**14 dB**	0,32	0,35	0,34	0,33	0,34	0,34	0,40
**12 dB**	0,39	0,42	0,43	0,41	0,44	0,48	0,53
**10 dB**	0,48	0,51	0,48	0,50	0,50	0,53	0,63
**8 dB**	0,56	0,60	0,61	0,62	0,64	0,73	0,84

Table S3. Top: calculated mean ε_{frame}; bottom: calculated standard deviation σ_{frame} for the results presented in Figure S3 in the supplements and Figure 9A in the manuscript.

2.4 Cross-correlation of the first derivative of the fitted Gamma Variate functions cut by method 1 - Figure 9B

Figure S4: Figure 9B in the manuscript: mean ε_{frames} using the Gamma Variate model on the data sets ($f_{sampling} = 25\,Hz$). Before fitting the model, cut method 1 is applied to the data set. To obtain the transit time the cross-correlation of the first derivative is computed.

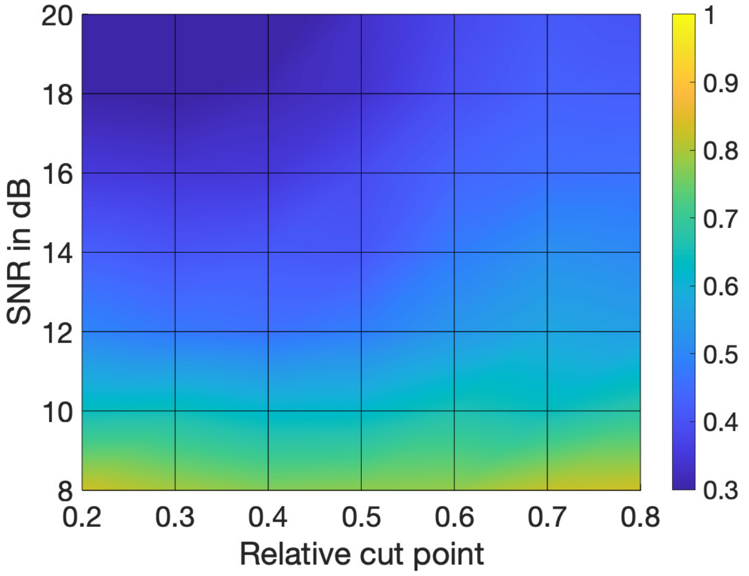

μ in frames							
	Cut level in % of C_{max}						
**SNR**	**20%**	**30%**	**40%**	**50%**	**60%**	**70%**	**80%**
**20 dB**	0,27	0,27	0,28	0,33	0,38	0,42	0,40
**18 dB**	0,30	0,29	0,32	0,34	0,40	0,43	0,43
**16 dB**	0,35	0,34	0,35	0,39	0,44	0,46	0,46
**14 dB**	0,43	0,41	0,42	0,41	0,48	0,52	0,51
**12 dB**	0,50	0,47	0,46	0,48	0,53	0,57	0,55
**10 dB**	0,64	0,65	0,62	0,62	0,66	0,64	0,68
**8 dB**	0,83	0,80	0,77	0,78	0,78	0,82	0,83
**σ in frames**							
**20 dB**	0,17	0,18	0,17	0,19	0,21	0,24	0,22
**18 dB**	0,20	0,21	0,22	0,22	0,25	0,27	0,27
**16 dB**	0,26	0,24	0,24	0,26	0,28	0,30	0,32
**14 dB**	0,30	0,30	0,29	0,32	0,36	0,37	0,38
**12 dB**	0,38	0,36	0,36	0,36	0,39	0,41	0,42
**10 dB**	0,49	0,49	0,45	0,46	0,48	0,46	0,50
**8 dB**	0,61	0,60	0,57	0,58	0,62	0,63	0,61

Table S4. Top: calculated mean ε_{frame}; bottom: calculated standard deviation σ_{frame} for the results presented in Figure S4 in the supplements and Figure 9B in the manuscript.

2.5 Cross-correlation of the first derivative of the fitted LDRW functions cut by method 1 - Figure 9C

Figure S5: Figure 9C in the manuscript: mean ε_{frames} using the LDRW model on the data sets ($f_{sampling} = 25\,Hz$). Before fitting the model, cut method 1 is applied to the data set. To obtain the transit time the cross-correlation of the first derivative is computed.

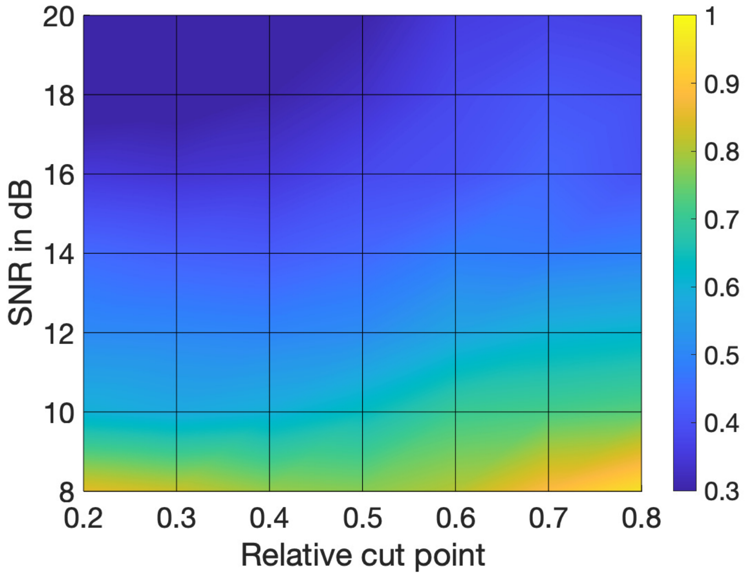

μ in frames							
	Cut level in % of C_{max}						
**SNR**	**20%**	**30%**	**40%**	**50%**	**60%**	**70%**	**80%**
**20 dB**	0,26	0,25	0,25	0,30	0,36	0,37	0,36
**18 dB**	0,27	0,28	0,30	0,33	0,38	0,41	0,40
**16 dB**	0,35	0,34	0,35	0,38	0,40	0,44	0,40
**14 dB**	0,45	0,43	0,42	0,44	0,48	0,48	0,49
**12 dB**	0,51	0,50	0,50	0,51	0,57	0,60	0,62
**10 dB**	0,60	0,58	0,60	0,64	0,71	0,72	0,74
**8 dB**	0,85	0,83	0,78	0,77	0,81	0,89	0,96
**σ in frames**							
**20 dB**	0,16	0,17	0,18	0,19	0,21	0,23	0,22
**18 dB**	0,18	0,19	0,20	0,21	0,24	0,27	0,28
**16 dB**	0,25	0,23	0,24	0,27	0,29	0,30	0,28
**14 dB**	0,32	0,31	0,30	0,32	0,38	0,39	0,35
**12 dB**	0,38	0,38	0,38	0,37	0,43	0,45	0,46
**10 dB**	0,48	0,46	0,46	0,46	0,50	0,51	0,51
**8 dB**	0,60	0,59	0,57	0,60	0,65	0,66	0,72

Table S5. Top: calculated mean ε_{frame}; bottom: calculated standard deviation σ_{frame} for the results presented in Figure S5 in the supplements and Figure 9C in the manuscript.

3 RESULTS AT 60 FPS: FIGURES 10A - 11C IN THE MANUSCRIPT

3.1 Cross-correlation of the raw data sets cut by method 3 - Figure 10A

Figure S6: Figure 10A in the manuscript: mean ε_{frames} using the raw data sets ($f_{sampling} = 60\,Hz$) with no mathematical fits. To obtain the transit time the cross-correlation is computed after applying the cut method 2.

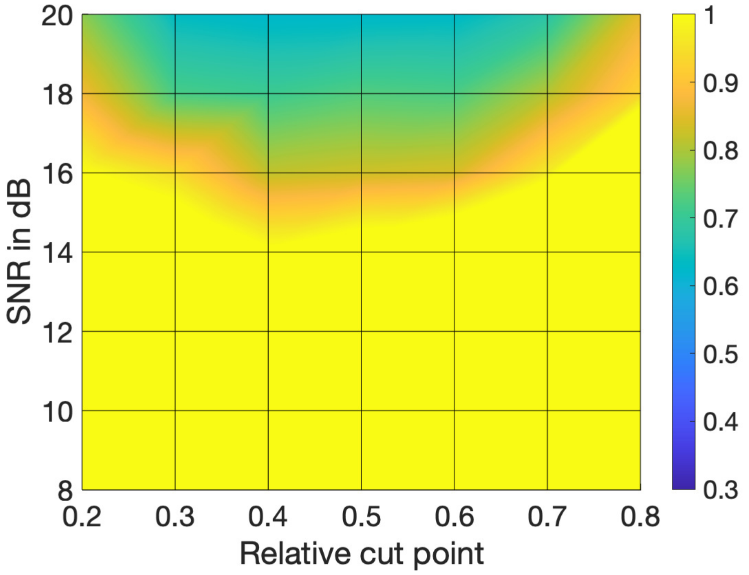

μ in frames

SNR	Cut level in % of C_{max} 20%	30%	40%	50%	60%	70%	80%
**20 dB**	0,78	0,63	0,62	0,62	0,63	0,68	0,85
**18 dB**	0,87	0,72	0,71	0,73	0,72	0,81	0,93
**16 dB**	1,01	0,94	0,82	0,83	0,84	0,97	1,30
**14 dB**	1,17	1,11	1,01	1,07	1,14	1,30	1,62
**12 dB**	1,56	1,37	1,27	1,25	1,20	1,40	1,78
**10 dB**	1,78	1,65	1,52	1,42	1,61	1,81	2,31
**8 dB**	2,19	2,04	1,88	1,86	2,00	2,19	2,78

σ in frames

SNR	20%	30%	40%	50%	60%	70%	80%
**20 dB**	0,54	0,45	0,40	0,42	0,41	0,47	0,60
**18 dB**	0,61	0,49	0,48	0,47	0,52	0,55	0,64
**16 dB**	0,71	0,69	0,59	0,63	0,60	0,74	0,90
**14 dB**	0,88	0,82	0,78	0,82	0,83	0,93	1,17
**12 dB**	1,19	1,10	0,98	0,96	0,96	1,11	1,33
**10 dB**	1,34	1,26	1,17	1,07	1,16	1,30	1,74
**8 dB**	1,62	1,56	1,48	1,42	1,54	1,64	2,06

Table S6. Top: calculated mean ε_{frame}; bottom: calculated standard deviation σ_{frame} for the results presented in Figure S6 in the supplements and Figure 10A in the manuscript.

3.2 Cross-correlation of the interpolated data sets cut by method 2 - Figure 10B

Figure S7: Figure 10B in the manuscript: mean ε_{frames} using the linearly interpolated data sets ($f_{sampling} = 60 \cdot 100\, Hz$) with no mathematical fits. To obtain the transit time the cross-correlation is computed after applying the cut method 2.

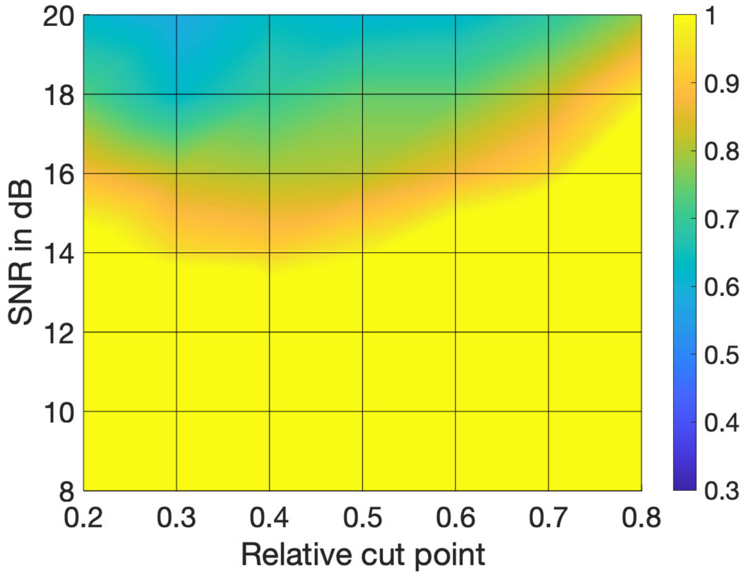

μ in frames							
	Cut level in % of C_{max}						
**SNR**	**20%**	**30%**	**40%**	**50%**	**60%**	**70%**	**80%**
**20 dB**	0,62	0,58	0,63	0,61	0,62	0,68	0,78
**18 dB**	0,73	0,63	0,70	0,74	0,74	0,82	0,97
**16 dB**	0,88	0,82	0,80	0,81	0,87	0,93	1,19
**14 dB**	1,06	0,95	0,92	0,98	1,11	1,25	1,46
**12 dB**	1,30	1,28	1,16	1,22	1,31	1,46	1,88
**10 dB**	1,67	1,44	1,39	1,52	1,62	1,85	2,30
**8 dB**	2,05	1,88	1,74	1,76	1,96	2,28	2,89
**σ in frames**							
**20 dB**	0,43	0,39	0,43	0,41	0,43	0,48	0,56
**18 dB**	0,55	0,49	0,48	0,50	0,52	0,57	0,68
**16 dB**	0,64	0,56	0,59	0,57	0,58	0,69	0,88
**14 dB**	0,83	0,71	0,72	0,75	0,82	0,93	1,14
**12 dB**	1,03	0,99	0,92	0,96	1,00	1,11	1,37
**10 dB**	1,26	1,15	1,10	1,14	1,21	1,40	1,73
**8 dB**	1,61	1,39	1,31	1,32	1,45	1,71	2,17

Table S7. Top: calculated mean ε_{frame}; bottom: calculated standard deviation σ_{frame} for the results presented in Figure S7 in the supplements and Figure 10B in the manuscript.

3.3 Cross-correlation of the fitted Gamma Variate functions cut by method 3 - Figure 11A

Figure S8: Figure 11A in the manuscript: mean ε_{frames} using the Gamma Variate model on the data sets ($f_{sampling} = 60\,Hz$). Before fitting the model, cut method 3 is applied to the data set. To obtain the transit time the cross-correlation is computed.

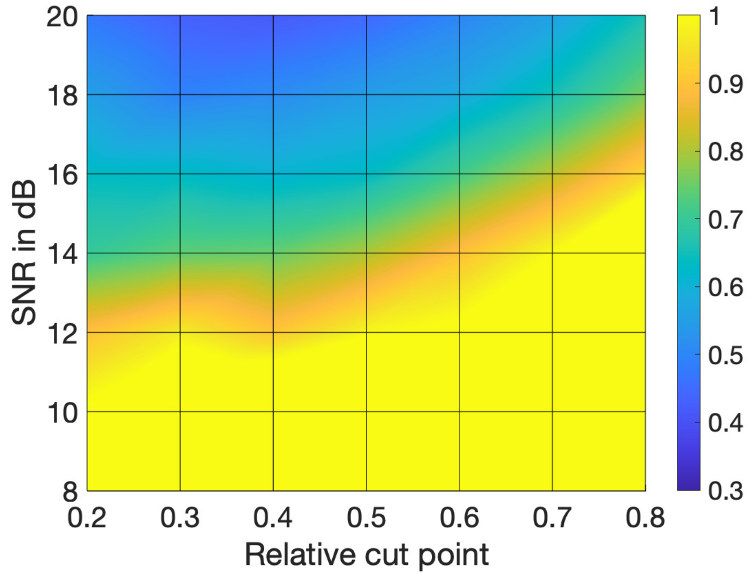

	**μ in frames**						
	**Cut level in % of C_{max}**						
**SNR**	**20%**	**30%**	**40%**	**50%**	**60%**	**70%**	**80%**
**20 dB**	0,47	0,43	0,42	0,44	0,50	0,56	0,66
**18 dB**	0,55	0,49	0,51	0,55	0,58	0,65	0,76
**16 dB**	0,63	0,63	0,61	0,63	0,72	0,81	0,94
**14 dB**	0,70	0,74	0,75	0,80	0,90	1,00	1,20
**12 dB**	0,90	0,97	0,91	0,99	1,02	1,18	1,48
**10 dB**	1,02	1,16	1,17	1,24	1,42	1,48	1,83
**8 dB**	1,34	1,39	1,45	1,58	1,65	1,92	2,33
	**σ in frames**						
**20 dB**	0,36	0,33	0,30	0,31	0,35	0,40	0,49
**18 dB**	0,41	0,36	0,36	0,41	0,42	0,47	0,59
**16 dB**	0,50	0,52	0,51	0,47	0,48	0,60	0,73
**14 dB**	0,52	0,64	0,54	0,59	0,66	0,75	0,95
**12 dB**	0,71	0,79	0,69	0,74	0,78	0,93	1,07
**10 dB**	0,79	0,95	0,85	0,92	1,01	1,10	1,39
**8 dB**	1,00	1,08	1,17	1,20	1,17	1,44	1,67

Table S8. Top: calculated mean ε_{frame}; bottom: calculated standard deviation σ_{frame} for the results presented in Figure S8 in the supplements and Figure 11A in the manuscript.

3.4 Cross-correlation of the fitted LDRW functions cut by method 3 - Figure 11B

Figure S9: Figure 11B in the manuscript: mean ε_{frames} using the LDRW model on the data sets ($f_{sampling} = 60\,Hz$). Before fitting the model, cut method 3 is applied to the data set. To obtain the transit time the cross-correlation is computed.

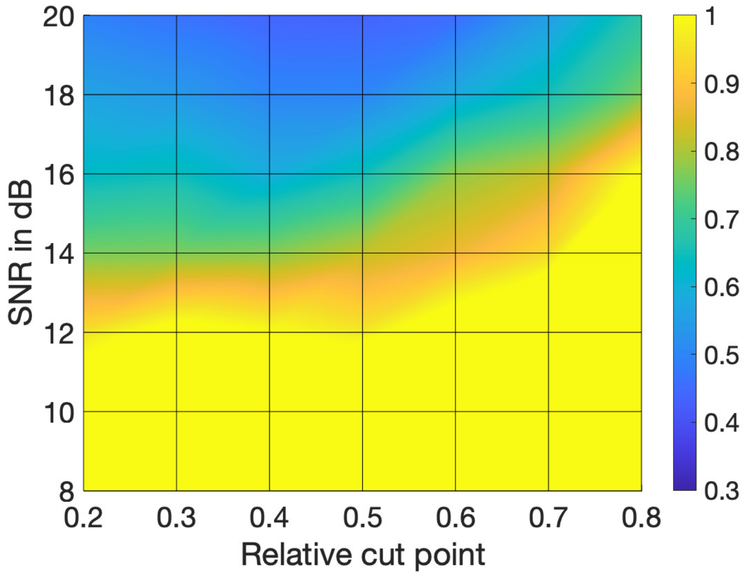

μ in frames							
	Cut level in % of C_{max}						
**SNR**	**20%**	**30%**	**40%**	**50%**	**60%**	**70%**	**80%**
**20 dB**	0,49	0,46	0,44	0,43	0,45	0,55	0,68
**18 dB**	0,56	0,54	0,50	0,50	0,58	0,65	0,76
**16 dB**	0,63	0,65	0,59	0,65	0,78	0,83	1,02
**14 dB**	0,76	0,77	0,77	0,82	0,87	0,94	1,27
**12 dB**	0,95	1,04	1,00	0,97	1,08	1,20	1,49
**10 dB**	1,10	1,15	1,18	1,29	1,48	1,60	1,85
**8 dB**	1,35	1,42	1,35	1,53	1,64	1,93	2,54
**σ in frames**							
**20 dB**	0,40	0,33	0,33	0,31	0,33	0,42	0,51
**18 dB**	0,45	0,41	0,37	0,35	0,42	0,46	0,58
**16 dB**	0,49	0,48	0,44	0,49	0,54	0,62	0,71
**14 dB**	0,59	0,66	0,61	0,61	0,64	0,71	0,91
**12 dB**	0,72	0,82	0,74	0,78	0,82	0,89	1,12
**10 dB**	0,86	0,94	0,91	0,99	1,10	1,23	1,34
**8 dB**	1,04	1,10	1,06	1,20	1,26	1,46	1,87

Table S9. Top: calculated mean ε_{frame}; bottom: calculated standard deviation σ_{frame} for the results presented in Figure S9 in the supplements and Figure 11B in the manuscript.

3.5 Cross-correlation of the first derivative of the fitted LDRW functions cut by method 1 - Figure 11C

Figure S10: Figure 11C in the manuscript: mean ε_{frames} using the LDRW model on the data sets ($f_{sampling} = 60\,Hz$). Before fitting the model, cut method 1 is applied to the data set. To obtain the transit time the cross-correlation of the first derivative is computed.

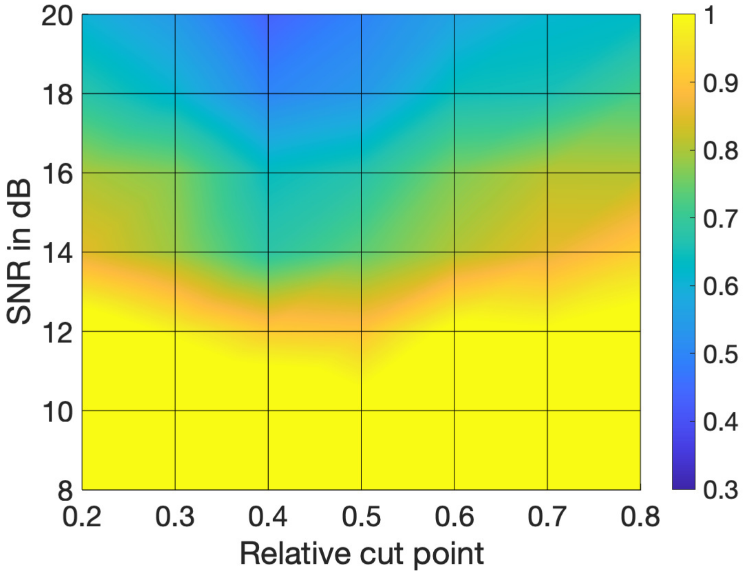

μ in frames							
	Cut level in % of C_{max}						
**SNR**	**20%**	**30%**	**40%**	**50%**	**60%**	**70%**	**80%**
**20 dB**	0,60	0,54	0,43	0,49	0,57	0,61	0,63
**18 dB**	0,68	0,61	0,50	0,54	0,65	0,66	0,72
**16 dB**	0,80	0,77	0,64	0,67	0,77	0,81	0,81
**14 dB**	0,85	0,79	0,68	0,74	0,80	0,86	0,92
**12 dB**	1,06	0,98	0,92	0,90	1,02	1,01	1,04
**10 dB**	1,16	1,14	1,11	1,04	1,16	1,20	1,27
**8 dB**	1,49	1,47	1,41	1,44	1,46	1,49	1,56
**σ in frames**							
**20 dB**	0,37	0,36	0,32	0,33	0,40	0,43	0,42
**18 dB**	0,44	0,43	0,38	0,39	0,44	0,47	0,51
**16 dB**	0,54	0,53	0,53	0,52	0,55	0,61	0,61
**14 dB**	0,59	0,58	0,57	0,58	0,63	0,65	0,67
**12 dB**	0,80	0,74	0,72	0,76	0,79	0,82	0,84
**10 dB**	0,86	0,84	0,82	0,86	0,88	0,95	0,92
**8 dB**	1,09	1,05	1,02	1,08	1,10	1,18	1,25

Table S10. Top: calculated mean ε_{frame}; bottom: calculated standard deviation σ_{frame} for the results presented in Figure S10 in the supplements and Figure 11C in the manuscript.

3.6 Expected change in morphology of an IDC in the setup

In this paper the IDCs are duplicated and shifted. This is justified by negligible morphological changes between two very close measurement points. The *in silico* model was used to clarify on the magnitude of changes. In contrast to the distances used in the main manuscript (intention there was to obtain IDCs with different morphology) we defined the distance to be 2 cm, which is expected in an *in vivo* setting. The obtained curves are shown in Figure S11. For a comparison of both curves the maximum value and the full width at half maximum (FWHM) were analyzed and are shown in Table S11. Thereby, the morphological changes measured by the maximum value and FWHM are below 0.5 $\%$.
To put this change into a relation to the performed investigation, we have applied noise to the signal to check whether the two sets of noisy IDCs can be distinguished. We used 1000 independent applications of white Gaussian noise at $20\,dB$ to the IDCs and their comparison showed no significance to distinguish the two curves using the two sided Mann-Whitney-U test ($p > 0.05$). This emphasizes that the morphological changes between the two measurement points are negligible.

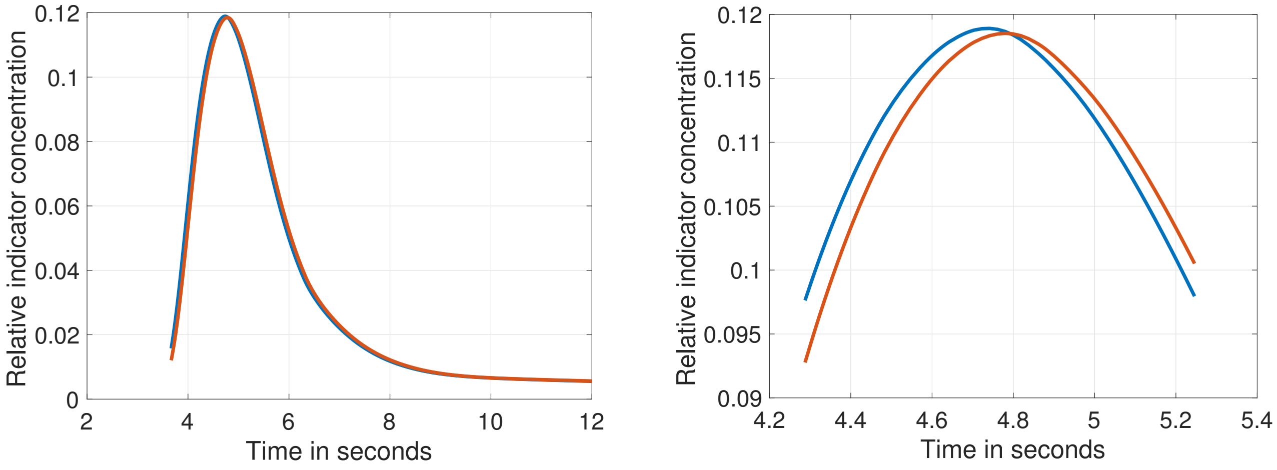

Figure S11: In blue the IDC at $50\,cm$; in red the IDC at $52\,cm$. To the left: the IDCs are shown and differences are barely visible. To the right: the section around the maximum of both IDCs is shown in detail. You can clearly see that the maximum is shifted in time which is expected. Very little differences are visible for the maximum value and the width of the IDCs. Detailed numbers are given in Table S11.

**Parameter**	IDC_{50cm}	IDC_{52cm}	Relative deviation
Maximum value	0.1189	0.1185	0.34%
FWHM in seconds	1.8325	1.8367	0.23%

Table S11. This table shows the maximum of the relative ICG concentration, the FWH and the relative deviation of two IDCs that were computed at $50\,cm$ and $52\,cm$ and their deviation.

3.7 Comparison of the simulation with measured IDC

The morphology of *in vivo* data varies a lot and is depending on many parameters such as distance of the measurement area to the injection site. So, depicting all possible *in vivo* settings is not possible and also not desirable. The purpose of this work is not to describe the nearly unlimited diversity in vascular geometry and fluid flow configuration. It shows that the use of mathematical functions to fit on IDCs has a large impact on the determined transit time. It was further shown that the statistical error is reduced using

those fits which will largely affect clinical flow rate measurement via fluorescence angiography (which is yet considered not applicable in cerebrovascular surgery).

Nevertheless, the subjective comparison of the presented *in vivo* and *in silivo* data sets (Figures 6A & B) show strong similarity (Pearson's correlation coefficient of 0.93). To underline this, we have recorded several *in vitro* data sets. The setup was presented by at a conference and the poster is attached to the supplementary material (Naber et al., 2019). Thereby, the proposed blood analog is used and pumped through a tube system to an area which is observed by a fluorescence microscope. A peristaltic pump provides the flow rate and an air trap is placed in the pipeline to smooth the peristaltic behavior of the pump. An industrial flow meter is used to measure the volume flow (Bronkhorst ES-Flow). The recorded IDCs are shown in Figures S12 to S14. The *in vitro* IDCs are shifted in time so their maximum is at the same position and the fluorescence intensity was scaled, so that the height match's the *in silico* data. They have a strong similarity with the *in silico* data set (Pearson's correlation coefficient of 0.98 - 0.99). Some deviations are present, which is not surprising since external factors (bending of the tube, trapped air bubbles, etc.) affect the experimental results.

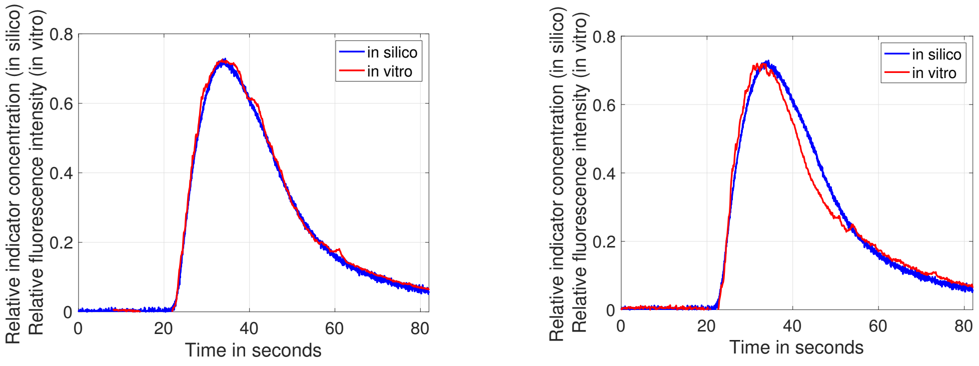

Figure S12

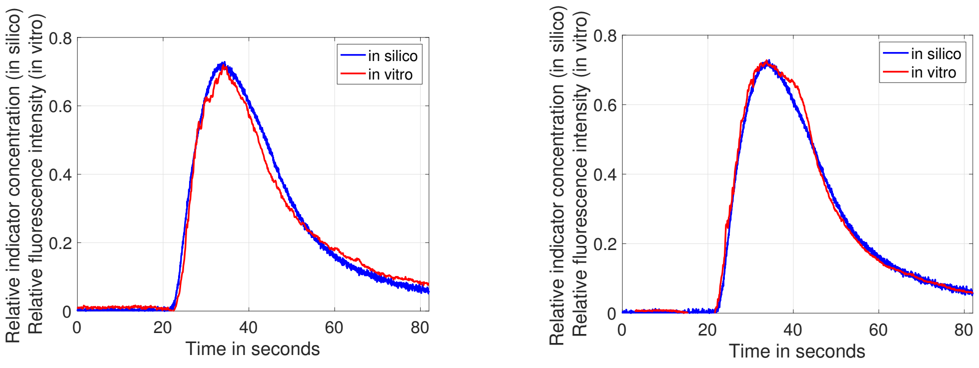

Figure S13

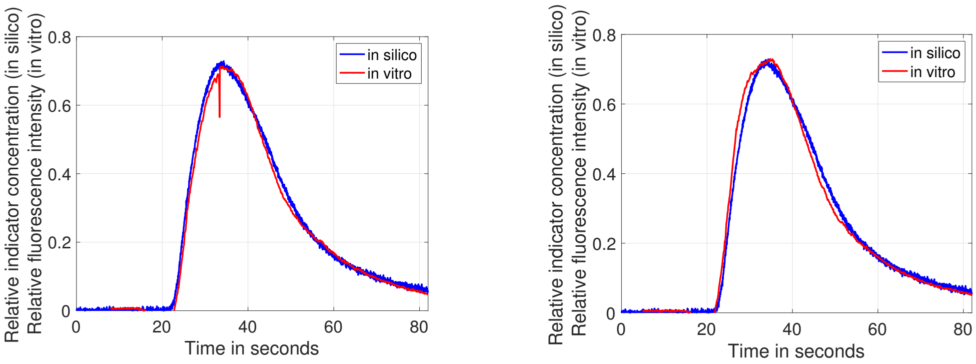

Figure S14

REFERENCES

Kinsella, J. E. and Whitehead, D. M. (1989). Proteins in whey: Chemical, physical, and functional properties. *Advances in Food and Nutrition Research* 33, 343–438

Naber, A., Meyer-Hilberg, L., and Nahm, W. (2019). Design of a flow phantom for the evaluation of quantitative ICG fluorescence angiography. In *Current Directions in Biomedical Engineering*. vol. 5

Tyn, M. T. and Gusek, T. W. (1990). Prediction of diffusion coefficients of proteins. *Biotechnology and Bioengineering* 35, 327–338. doi:10.1002/bit.260350402
